# The distribution of incubation and relapse times in experimental human infections with the malaria parasite *Plasmodium vivax*

**DOI:** 10.1186/1471-2334-14-539

**Published:** 2014-10-04

**Authors:** Andrew A Lover, Xiahong Zhao, Zheng Gao, Richard J Coker, Alex R Cook

**Affiliations:** Saw Swee Hock School of Public Health, National University of Singapore and National University Health System, Singapore, Singapore; Faculty of Engineering, National University of Singapore, Singapore, Singapore; Communicable Diseases Policy Research Group, London School of Hygiene and Tropical Medicine, Bangkok, Thailand; Yale-NUS College, National University of Singapore, Singapore, Singapore; Program in Health Services and Systems Research, Duke-NUS Graduate Medical School, Singapore, Singapore; Department of Statistics and Applied Probability, National University of Singapore, Singapore, Singapore; Communicable Diseases Centre, Tan Tock Seng Hospital, Singapore, Singapore

**Keywords:** Malaria, vivax, Infectious disease models, Incubation period, Relapse

## Abstract

**Background:**

The distributions of incubation and relapse periods are key components of infectious disease models for the malaria parasite *Plasmodium vivax*; however, detailed distributions based upon experimental data are lacking.

**Methods:**

Using a range of historical, experimental mosquito-transmitted human infections, Bayesian estimation with non-informative priors was used to determine parametric distributions that can be readily implemented for the incubation period and time-to-first relapse in *P. vivax* infections, including global subregions by parasite source. These analyses were complemented with a pooled analysis of observational human infection data with infections that included malaria chemoprophylaxis and long-latencies. The epidemiological impact of these distributional assumptions was explored using stochastic epidemic simulations at a fixed reproductive number while varying the underlying distribution of incubation periods.

**Results:**

Using the Deviance Information Criteria to compare parameterizations, experimental incubation periods are most closely modeled with a shifted log-logistic distribution; a log-logistic mixture is the best fit for incubations in observational studies. The mixture Gompertz distribution was the best fit for experimental times-to-relapse among the tested parameterizations, with some variation by geographic subregions. Simulations suggest underlying distributional assumptions have critically important impacts on both the time-scale and total case counts within epidemics.

**Conclusions:**

These results suggest that the exponential and gamma distributions commonly used for modeling incubation periods and relapse times inadequately capture the complexity in the distributions of event times in *P. vivax* malaria infections. In future models, log-logistic and Gompertz distributions should be utilized for general incubation periods and relapse times respectively, and region-specific distributions should be considered to accurately model and predict the epidemiology of this important human pathogen.

**Electronic supplementary material:**

The online version of this article (doi:10.1186/1471-2334-14-539) contains supplementary material, which is available to authorized users.

## Background

Malaria caused by *Plasmodium vivax* has recently entered the global health agenda in the context of global malaria elimination. This has followed a re-evaluation of the long-held opinion that this parasite causes limited morbidity and essentially no mortality; a range of recent studies suggest that it is a major contributor to both in the wide-spread regions where it is endemic [1, 2]. Furthermore, the presence of dormant liver forms (hypnozoites) which can re-activate infection is an important barrier in disease control towards global malaria elimination [3, 4].

Mathematical and statistical models are an important area of research in malaria given the complex dynamics of the parasite-host-vector system [5, 6]. The majority of malaria models have focused on the species common in sub-Saharan Africa, *P. falciparum*; only recently have efforts been directed towards *P. vivax*[7–10]. The distributions of event times like incubation period have an important role in modeling infectious disease [11], and realistic assumptions about the distributions are crucial for accurate models. Published *P. vivax* models have made a range of implicit and explicit assumptions about the functional form of incubation periods and relapse intervals with limited empirical justification. Earlier work has focused on statistically and clinically significant differences in the epidemiology of sub-populations of this parasite [12, 13], but these epidemiological models do not provide well-defined parametric distributions for application within mathematical or statistical models. The purpose of this study is to use data synthesis to provide accurate, realistic and readily implementable parameters for modeling *P. vivax* infection event times using several historical human infection datasets.

## Methods

We have utilized data from our earlier study of historical human challenge studies in two populations: patients receiving pre-antibiotic era neurosyphilis treatments, and prison volunteers in experiments for malaria prophylaxis. These two groups of institutionalized patients had mosquito-transmitted infections with defined exposure dates and complete follow-up [12]. Data for the infections with long-latency (extended incubation periods) were extracted from three published studies that involved two unrelated temperate strains; one involved drug prophylaxis [14], two were observational studies with inferred exposure dates [15, 16], and all include extensive interval censoring in reported event times.

**Figure 1:**
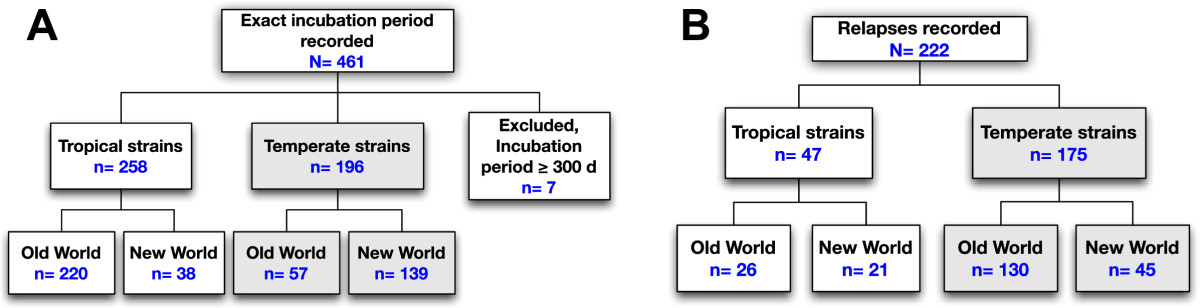
**CONSORT diagram for study populations,** ***Plasmodium vivax*** **malaria (A) experimental incubation period study (B) experimental time-to-first relapse study.**

The composition of the two populations for the incubation period analysis can be found in tables 1 and 2; the population for relapses is in Table 3. CONSORT diagrams for the two experimental studies can be found in Figure 1.

**Table 1:** **Study population for incubation period analysis (experimental studies)**

Characteristic		Number (%)
**Parasite origin**	New World, Temperate	139 (30.6)
	New World, Tropical	38 (8.4)
	Old World, Temperate	57 (12.6)
	Old World, Tropical	220 (48.5)
**Neurological treatment patient**	No	224 (49.3)
	Yes	230 (50.7)
**Total**		454

**Table 2:** **Study population for incubation period analysis (observational studies)**

Characteristic		Number (%)
**Malaria chemoprophylaxis**	No	109 (20.6)
	Yes	191 (36.1)
	Unknown	229 (43.3)
**Parasite strain**	Korean	262 (49.5)
	St. Elizabeth	267 (50.5)
**Total**		529

**Table 3:** **Study population for relapse period analysis (experimental studies)**

Characteristic		Number (%)
**Parasite origin**	New World, Temperate	45 (20.3)
	New World, Tropical	21 (9.5)
	Old World, Temperate	130 (58.6)
	Old World, Tropical	26 (11.7)
**Neurological treatment patient**	No	87 (39.2)
	Yes	135 (60.8)
**Total**		222

Individuals without a recorded incubation or relapse (censored observations) have not been included in this analysis. ‘Failed’ primary infections were generally not reported within the original studies and may represent experimental difficulties. In analysis of relapses, our primary consideration was to determine the underlying distribution of events for modeling; non-parametric ding the proportion with relapses, can be found in the Additional file 1 (section III).

Case-patients were exposed to parasites from a range of geographic locations, which were characterized by hemisphere and latitude. As in prior studies and historical precedence, the sub-populations from the Western hemisphere are referred to as the New World, and Old World region consists of the Eastern hemisphere and Pacific regions [17]; temperate and tropical regions have been split at ± 27.5° N/S. Many of these data include interval censoring; that is, the event was reported as occurring within a specified time interval, but the exact time in unknown [18].

This study analyzes de-identified, secondary data published in the open literature (in the public domain); no ethics review was required. Analysis of data from patients at the same neurosyphilis treatment centers has been published with an extensive discussion of the ethical issues [19]; the issues inherent to the prison volunteers in these studies have also received extensive attention [20–22].

**Figure 2:**
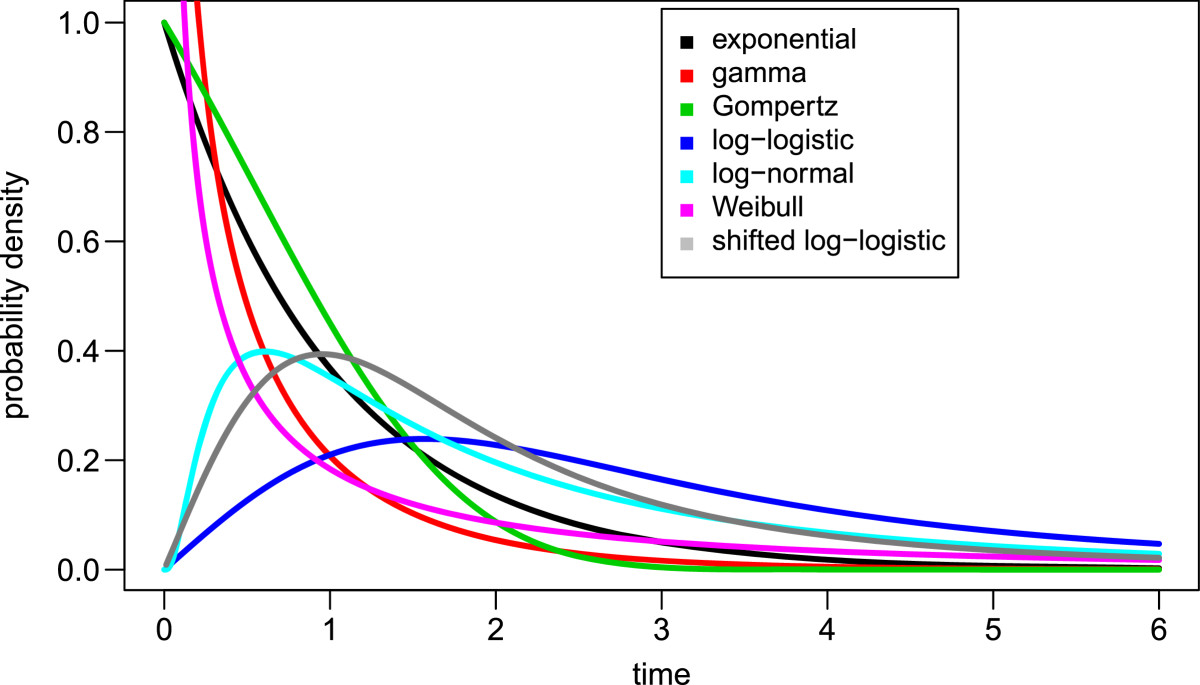
**Comparison of general probability distributions included within this study [shape = 0.5, rate = 1; shift = 0.5 (shifted log-logistic only)].**

The incubation period refers to the time from parasite exposure to onset of clinical symptoms; prepatent periods, which refer to the identification of blood-stage parasites, were not included in this analysis. For the experimental studies, all patients received only symptomatic treatments; all cases with malaria prophylaxis or radical cure were excluded. Relapses were measured from the primary infection as reported by the original study authors, and correspond to the onset of new clinical symptoms after parasites are no longer visible in the peripheral blood following the primary infection [23]. These data were examined using survival models, to specifically address the non-normal distribution of event times.In this analysis, we examined a range of distributions including exponential, gamma, Gompertz, log-logistic, log-normal, Weibull; time-shifted distributions from these respective families; and mixture distributions from each distribution family. The general forms of these distributions are shown in Figure 2.

Model fitting and parameter estimation utilized the Markov-Chain Monte-Carlo algorithm [24], and interval censored data were addressed using data augmentation methods [25]. Two complementary sets of analyses were performed for each of the experimental incubation and relapse datasets. In the first set, the best-fit distribution was found using the aggregate data, and then parameters for this optimal distribution were determined for each of the subregions of interest. In the second analysis, the best-fit distribution was found for each of the subregions independently.

Deviance Information Criterion (DIC) was used for model comparison [26], with standard thresholds to determine strength of evidence. That is, an absolute difference between models of < 2 DIC units was taken as indicating little difference; from 2-7 units indicating large differences; and > 7 DIC units indicating clear evidence of superiority.

To examine the sensitivity of the model selection procedure, we multiplied all time points by log-normal noise, with mean of 0 on the log scale, plus 0.01 standard deviation, i.e. randomly scaled up or down by ± 2%. Model sensitivity was then assessed by comparing the DIC from best-fit model with the DIC value from fitting the same distributional model to the generated pseudodata.

To assess the epidemiological and practical impacts of identified distributions, we performed a series of stochastic compartmental (SIR) models at fixed R_0_ values while varying the underlying distributions. The distributions were implemented using the best-fit parameters from our data augmentation process. For these epidemic simulations, we utilized the R0 package in R [27] for discrete-time models, running 10,000 stochastic simulations, reporting the mean values for each of the resulting sets of epidemics. We have not incorporated uncertainty in the extrinsic incubation period due to lack of reliable data, and we have made the assumption that the incubation period distribution is proportional to the generation interval, as *P. vivax* infections produce infective gametocytes rapidly upon onset of clinical symptoms [28].

We used non-informative priors for all parameter estimations. Proposal distributions were adjusted using estimated means and covariances from pilot runs in an iterative process to accelerate convergence; assessment of convergence was performed using Geweke’s diagnostic [29]. All statistical analyses were performed in R (version 2.15.2) [30], using the packages fitdistrplus, flexsurv, grid, MASS, seqinr, FAdist, stats4, R0 and custom-built code for the MCMC algorithm.

## Results

### Incubation periods

**Figure 3:**
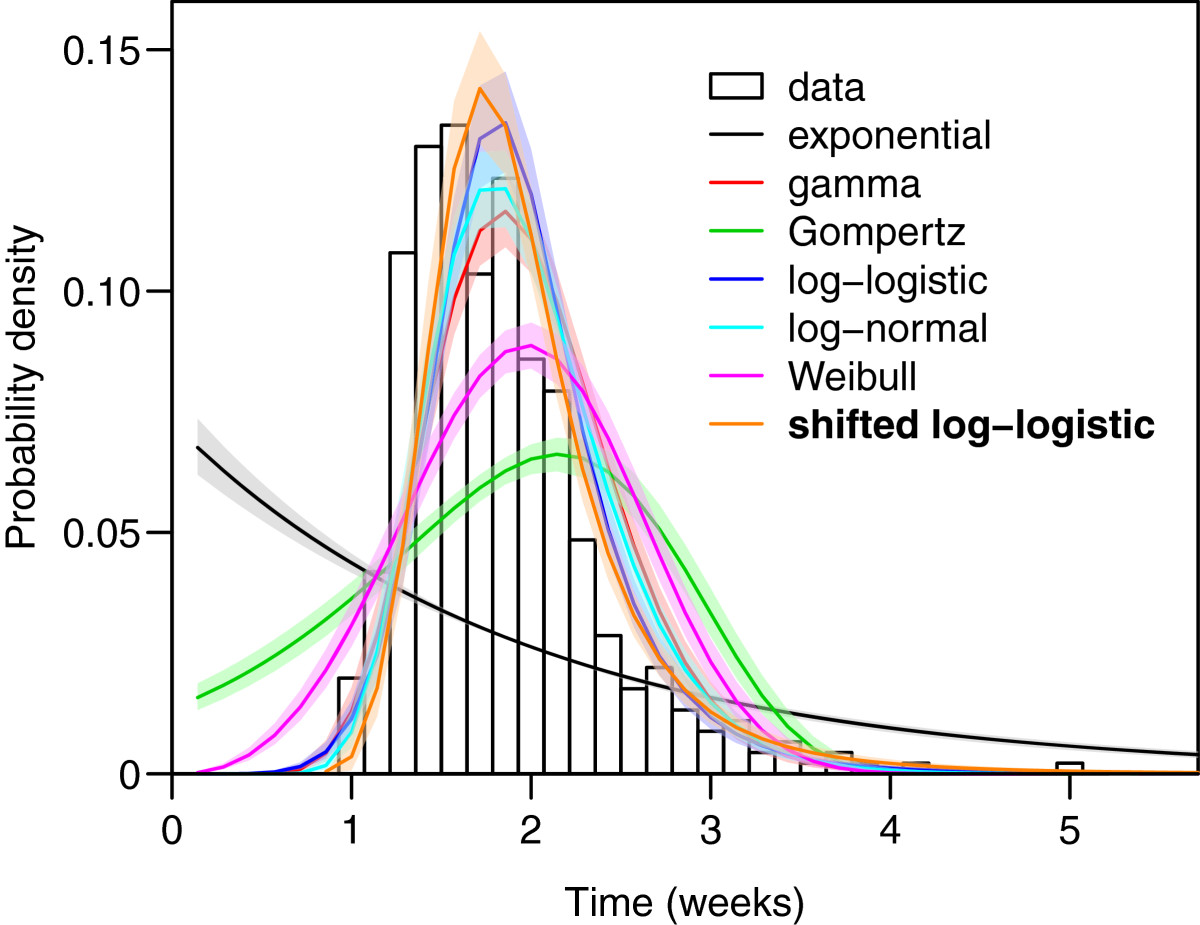
**Comparison of crude (non-data augmented) data and estimated parametric models of experimental incubation times,** ***Plasmodium vivax*** **malaria (N = 454).** Experimental data are in black outlines, and parametric model fits are shown with 95% confidence intervals, along with the overall best parametric fit.

The study of experimental incubation period included 461 case-patients and overlaid distributions can be found in Figure 3; DIC comparisons for these distributional families for both the aggregate and for subregion-specific incubation period data are shown in Table 4. It should be noted for all the results that ‘flattening’ of the fitted curves relative to the data-based histograms arises from the data augmentation processes. The Deviance Information Criterion (DIC) indicates that the shifted log-logistic distribution has a substantially better fit than the second best shifted log-normal distribution, as evidenced by a DIC difference of 0.4. Mixture distributions of two gammas had limited levels of support (Δ DIC < 3), while all other distributions were not supported by DIC. The increased complexity of mixture distributions does not explain any greater variation in these data, and are also not supported by DIC. The subregion specific distributions show some differences from the best-fit distribution (shifted log-logistic) from the aggregate data. Among the New World, tropical parasites there is support for a shifted Gompertz distribution (Δ DIC = 2.5), and in the New World, temperate strains there is very strong evidence for a shifted Weibull distribution (Δ DIC = 18.3). Quadrant-specific plots of Kaplan-Meier curves with best-fit distributions can be found in the Additional file 1 (section II).

**Table 4:** **Fitted distributions for experimental incubation times,** ***Plasmodium vivax*** **malaria**

Distribution	Old World, Tropical	Old World, Temperate	New World, Tropical	New World, Temperate	Global fit, All regions
	∆ DIC	∆ DIC	∆ DIC	∆ DIC	∆ DIC
Exponential	583.1	111.8	109.8	332.9	917.2
Shifted exponential	114.3	45.5	1.8	141.5	275.0
Mixture exponential	-	-	-	-	919.1
Weibull	76.2	3.7	1.6	0.1	166.4
Shifted Weibull	12.3	2.3	0.2	**0.0**	33.7
Mixture Weibull	-	-	-	**-**	40.1
Log-normal	4.7	4.1	1.0	15.4	16.3
Shifted log-normal	2.5	5.5	1.1	17.6	0.4
Mixture log-normal	-	-	-	-	18.3
Log-logistic	3.1	**0.0**	3.4	16.6	14.2
**Shifted log-logistic**	**0.0**	1.5	2.5	18.3	**0.0**
Mixture log-logistic	**-**	-	-	-	16.1
Gamma	11.0	1.3	0.6	9.2	40.3
Shifted gamma	6.5	1.6	1.4	11.1	12.5
Mixture gamma	-	-	-	-	2.7
Gompertz	159.4	17.3	0.8	10.6	366.0
Shifted Gompertz	42.6	15.5	**0.0**	4.9	142.1
Mixture Gompertz	-	-	**-**	-	100.7
**DIC of best-fit model**	958.8	345.5	163.6	680.1	2374.6

Note: mixture models showed non-normal posteriors and are not reported.

Best fitting distributions are shown in bold.

**Figure 4:**
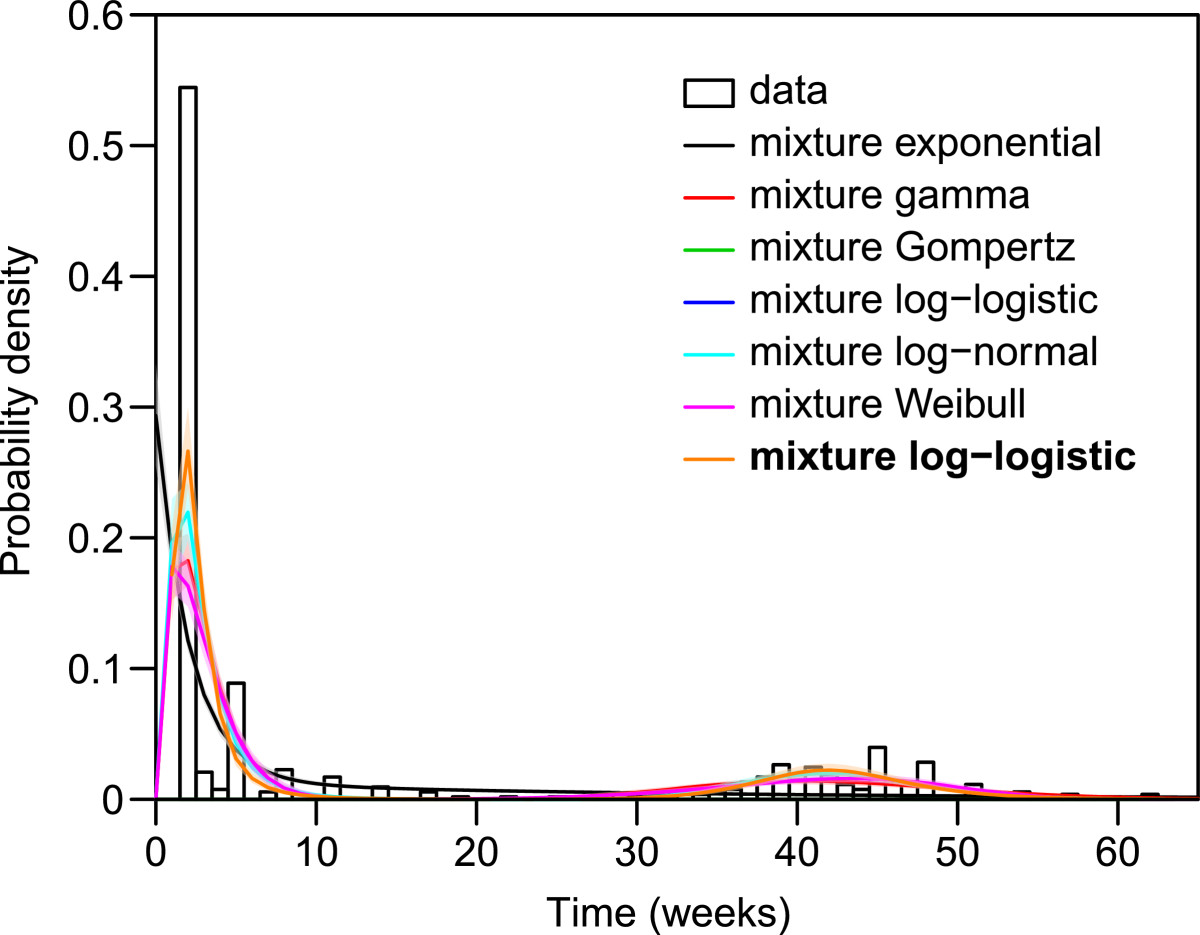
**Comparison of crude (non-data augmented) data and estimated parametric model of observational incubation times,** ***Plasmodium vivax*** **malaria (N = 529).** Observational data are in black outlines, and parametric model fits are shown with 95% confidence intervals, along with the overall best parametric fit.

The distribution of the 529 cases in confounded and observational studies with longer-term incubations is shown in Table 5 and Figure 4; a bimodal peak is evident. The studies with the St. Elizabeth strain involved a range of chemoprophylaxis regimens, and the Korean strain infections were all observational studies that largely included chemoprophylaxis. These results also overwhelmingly support a log-logistic distribution; in this case a mixture of two log-logistic distributions accurately capture the bimodal distribution commonly observed in temperate zone epidemiology. Shifted distributions showed extremely poor fit and are not reported. A Kaplan-Meier curve for these data can be found in the Additional file 1 (section II).

**Table 5:** **Fitted distributions for observational incubation time studies,** ***Plasmodium vivax*** **malaria**

Standard distributions		Mixture distributions	
Base model	∆ DIC	Base model	∆ DIC
exponential	952.4	exponential	551.5
gamma	570.6	gamma	171.8
Gompertz	874.2	Gompertz	297.7
log-logistic	719.3	**log-logistic**	0.0
log-normal	688.4	log-normal	41.5
Weilbull	611.9	Weilbull	193.4
DIC of best-fit model			**2876.7**

(Note: shifted distributions showed extremely poor fit and are not reported).

The best fitting distribution is shown in bold.

### Times to first relapse

**Figure 5:**
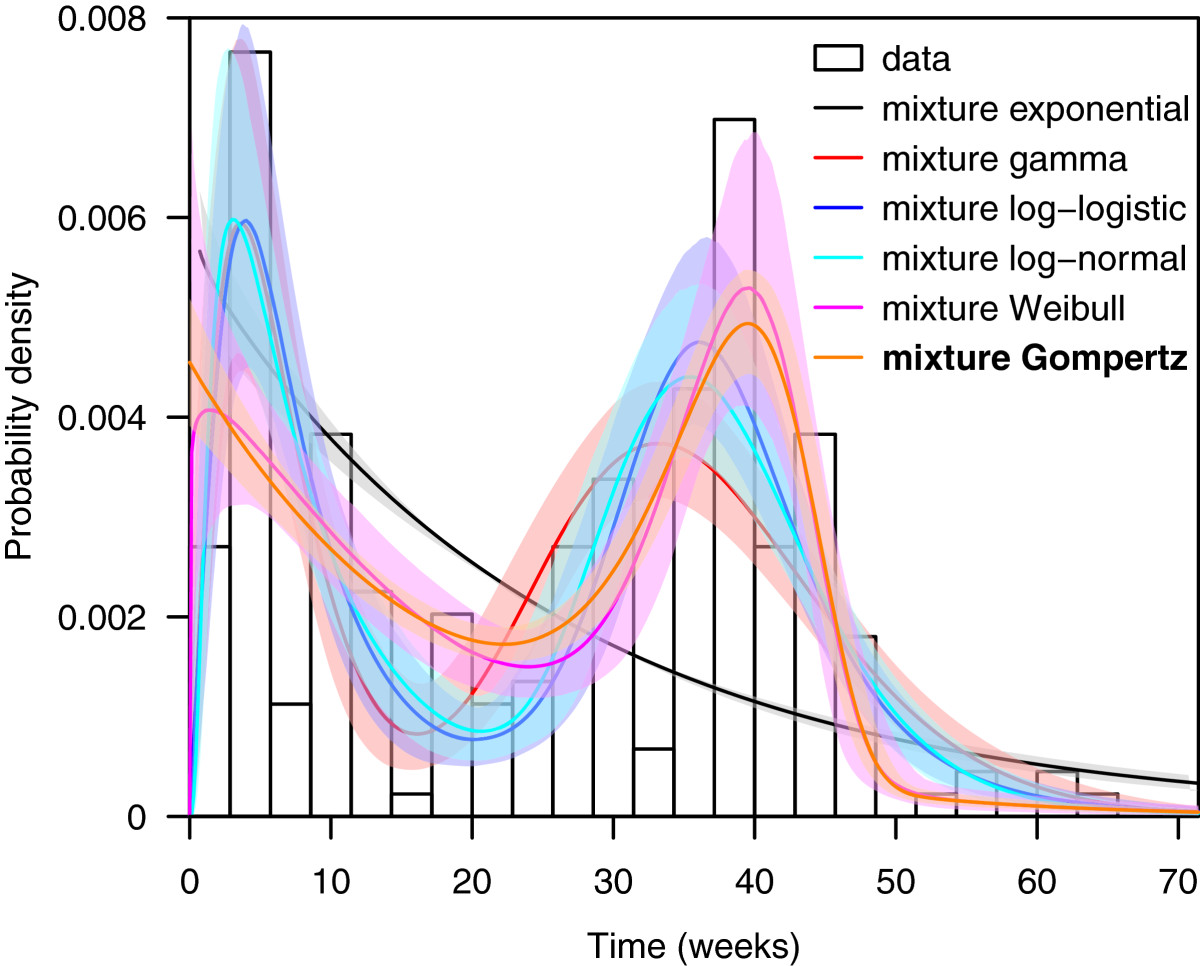
**Comparison of crude (non-data augmented) data and estimated parametric model of first relapse times,** ***Plasmodium vivax*** **malaria.** (N = 222). Experimental data are in black outlines, and parametric model fits are shown with 95% confidence intervals, along with the best overall parametric fit.

The results of the time-to-relapse analysis (primary infection to the first relapse) are shown in Table 6. We find that mixture distributions provide better fit for the total dataset than standard families; specifically, we find the best fit with a Gompertz mixture, followed by the log-logistic mixture (Δ DIC = 4.1), log-normal mixture (Δ DIC = 4.7) and Weibull mixture (Δ DIC = 5.4). While the differences among these three distributions are very minor, all capture the event times poorly relative to the Gompertz. The gamma and exponential mixtures both fit poorly (Δ DIC > 7). Figure 5 shows these distributions compared with the experimental data; the district bimodal peak is captured by the Gompertz mixture. There is some limited support for a shifted Gompertz in the New World Tropical region (Δ DIC = 2.9), but the remaining regions, and the global fit to aggregate data all show strong statistical support for a mixture Gompertz distribution.

**Table 6:** **Fitted distributions for experimental relapse times,** ***Plasmodium vivax*** **malaria**

Distribution	Old World, Tropical	Old World, Temperate	New World, Tropical	New World, Temperate	Global fit, All regions
	∆ DIC	∆ DIC	∆ DIC	∆ DIC	∆ DIC
Exponential	112.3	121.8	39.0	106.2	147.4
Shifted exponential	86.2	80.1	13.3	103.9	134.1
Mixture exponential	109.1	121.1	40.2	106.4	144.9
Weibull	138.1	221.2	111.8	212.4	190.7
Shifted Weibull	55.6	79.2	13.2	104.5	120.0
Mixture Weibull	80.5	3.8	4.2	14.3	5.4
Log-normal	98.5	111.1	9.0	126.4	178.9
Shifted log-normal	60.4	151.0	34.3	146.6	219.8
Mixture log-normal	75.4	13.6	11.1	17.5	4.7
Log-logistic	93.7	125.2	8.2	121.4	189.3
Shifted log-logistic	60.5	131.9	25.2	129.8	190.1
Mixture log-logistic	94.5	126.8	9.5	122.3	4.1
Gamma	112.0	94.3	9.7	104.7	130.5
Shifted gamma	54.3	80.8	15.0	105.5	131.0
Mixture gamma	86.4	25.7	9.3	1.1	7.5
Gompertz	114.0	107.8	2.7	64.1	74.9
Shifted Gompertz	89.4	79.1	**0.0**	63.9	73.4
**Mixture Gompertz**	**0.0**	**0.0**	2.9	**0.0**	**0.0**
**DIC of best-fit model**	144.5	1402.0	219.6	467.17	2596.2

Best fitting distributions are shown in bold.

### Sensitivity analyses

A sensitivity analysis was performed for all three datasets and each of the subregions individually, and show strong evidence that the models provided good fits for the pseudodata by comparisons of the DIC values. Detailed results, estimated posterior distributions for model parameters overall and by quadrant, are presented in the Additional file 1.

### Epidemic simulations

**Figure 6:**
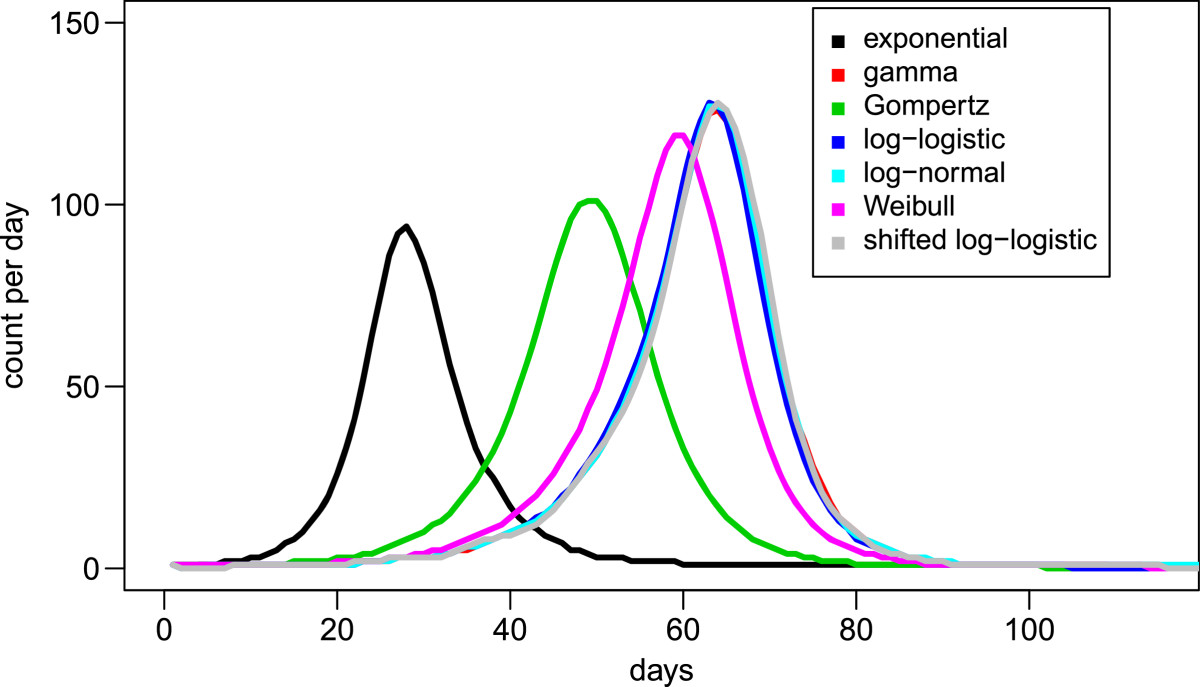
**Comparison of simulated** ***Plasmodium vivax*** **malaria epidemics with R**_**0**_ **= 5 (mean values from 10,000 simulations for each standard distribution).**

The results of stochastic epidemic simulations can be found in Figure 6. These results suggest that at a reproductive number (R_0_) of 5, the time scale of a modeled epidemic varies dramatically based upon the distribution of the incubation period. Use of an exponential distribution, as is extremely common in SIR compartmental simulations, shows a much more rapid epidemic, with Gompertz and Weibull distributions showing more gradual epidemic evolution. Finally, gamma, log-logistic, log-normal, and shifted log-logistic have the latest epidemic peaks, and are virtually indistinguishable from one another. The mean total cases for each set of 10,000 simulations by underlying distributions are shown in Table 7. Comparison of these totals shows that within the 95% confidence intervals, the total number of cases within the epidemic is greater for the best-fitting log-logistic and shifted log-logistic distributions relative to exponential- and gamma-distributed incubation periods. Simulations with R_0_ = 50 and 75 produced consistent results but with greater separation of the gamma, log-logistic, log-normal, and shifted-log-logistic epidemic curves (results not shown).

**Table 7:** **Total case counts from epidemic simulations,** ***Plasmodium vivax*** **malaria (mean values and 95% CIs from 10,000 simulations for each distribution)**

Distribution	Total case count	(95% CI)
exponential	1333	(1330 to 1335)
gamma	2329	(2326 to 2332)
Gompertz	1950	(1947 to 1953)
log-logistic	2306	(2304 to 2310)
log-normal	2328	(2325 to 2331)
Weilbull	2216	(2214 to 2220)
shifted log-logistic	2330	(2328 to 2334)

## Discussion

Although some of the earliest simulation models of malaria were directed towards *P. vivax* in epidemics, this parasite has received limited attention from modelers [31].

The models that have appeared have used a range of distributions for the model parameters of incubation period and time-to-relapse. Some of the earliest comprehensive mathematical models for *P. vivax* did not consider distributional assumptions and relied on point estimates [32]; other mathematical models used a log-normal distribution for relapses and a single estimate of 15 days for incubation period [7], implying an exponential distribution. A stochastic model of potential *P. vivax* transmission within Japan used a gamma distribution for the incubation period, an exponential distribution for short relapse periods, and a log-normal for longer relapses [33].

Two other comprehensive mathematical models implicitly assume exponential distributions for both incubation and times-to-relapse [9, 34]. A recent comprehensive model including multiple relapse states used a 15-day incubation period in simulations to produce a mean relapse interval of 7.1 months for cases in India, with incubation as an exponential distribution, and relapses modeled using a gamma distribution [10].

Several studies have found that results from infectious disease models can be highly sensitive to accurate distributional assumptions [35, 36]; our study reinforces these conclusions in finding the ‘default’ exponential and gamma distributions, used for mathematical tractability, inadequately capture the complexity of experimental data [37, 38]. The results from our simulations concur with these statements and suggest that use of best-fitting distributions can lead to larger overall case-counts and slower epidemic evolution than would be predicted based upon exponential or gamma distributed incubation periods. As the underlying distributional assumptions have large and important impacts upon both the time-scale of epidemic evolution and total case counts in *P. vivax* epidemics, these parameters are therefore a critical component of accurate models.

A range of entomological, molecular, genetic, and epidemiological evidence suggests the existence of subspecies within *P. vivax*[12, 17, 39]; however there has been limited consideration of this aspect of parasite biology in published models [9]. Little empirical data exists to support models that include explicit consideration of this aspect of the epidemiology; this study provides parameterization for subpopulations by climactic zone (temperate and tropical) as well as the postulated subspecies *P. vivax vivax* (E. hemisphere) and *P. vivax collinsi* (W. hemisphere) to inform region-based models towards global malaria elimination. Our results show that shifted log-logistic distributions adequately capture the incubation period for all regions except for the New World, temperate parasites, which show strong support for a shifted Weibull. However, as these parasite populations were eliminated in the early 20^th^ century, they have limited relevance for modern modeling studies [40].

The results from the observational long-latent infections have several important implications. Although the biological underpinnings of relapse remain obscure [41], there has been considerable debate that long-latencies may in fact be relapses after a sub-clinical primary infection [42]. The results from this study show that relapses exhibit quantitatively different behavior at a population-level from these long-incubation periods, and this in turn suggests a closer biological link to ‘normal’ incubations than to relapses.

Secondly, the congruence of the distributions from experimental and observational studies suggests that results from observational studies, while inherently limited, may still adequately capture the natural history of infection with *P. vivax.* This finding may greatly expand the utility of available datasets to more completely explore the epidemiology of *P. vivax*.

In modeling the time-to-relapse, there is strong support for a mixture Gompertz for all event times except in the New World Tropical region, where a shifted Gompertz is supported. In addition to simplifying modeling, this concordance of distributions in different parasite populations suggests that hypnozoite activation may have a common underlying biological trigger, regardless of parasite genetics [43]. While these results for temperate zone parasites are based primarily on now-eliminated Russian strains, the parasites currently circulating on the Korean peninsula have been reported to have similar relapse patterns [3].

However, this study has several limitations. The times we have analyzed are from adult, non-immune and mostly Caucasian subjects with uncertain inclusion or exclusion criteria, and may not represent the experience in high transmission settings due to the influence of immunological factors, as well as the poorly understood impact of mixed-species malaria infections [44]. While malariotherapy for neurosyphilis treatment has been shown to have minimal impacts on incubation periods, larger impacts were found for relapse periods in some sub-populations of *P. vivax*[12].

A related study examined the length of *P. falciparum* infections found that the total duration of infections were best modeled using a Gompertz distribution [37]. However, the existence of relapses makes defining a duration of infection with *P. vivax* difficult; multiple lines of evidence suggests that relapses within a single infection may be genetically distinct from the primary infection [45].

## Conclusions

Our results suggest that the ‘default’ distributions used in many modeling studies (exponential and gamma distributions), may be inadequate to fully capture the natural variability and complexity of event times in human infections with *Plasmodium vivax* malaria. Future modeling studies should consider the use of log-logistic and Gompertz distributions for incubation periods and relapse times respectively, and the region-specific distributions included in this work should be considered to accurately model regional variations in the epidemiology of this parasite. Future statistical and mathematical models of *P. vivax* transmission should incorporate the more complex distributions identified in this study to maximize the congruence with the true natural history and epidemiology of this important human pathogen.

## Electronic supplementary material

Additional file 1: Contains detailed posterior distributions and sensitivity analyses from this study; failure proportions and Kaplan-Meier plots for relapse studies; and detailed parameterization of distributions.(DOCX 832 KB)

Below are the links to the authors’ original submitted files for images.

Authors’ original file for figure 1

Authors’ original file for figure 2

Authors’ original file for figure 3

Authors’ original file for figure 4

Authors’ original file for figure 5

Authors’ original file for figure 6
