# Supplementary material for: The distribution of incubation and relapse times in experimental human infections with the malaria parasite Plasmodium vivax
Source: BMC Infect Dis. 2014 Oct 4;14:539. doi: 10.1186/1471-2334-14-539 (PMC4287165; doi:10.1186/1471-2334-14-539)
Supplement: Supplementary file 1 — Additional file 1: Contains detailed posterior distributions and sensitivity analyses from this study; failure proportions and Kaplan-Meier plots for relapse studies; and detailed parameterization of distributions.(DOCX 832 KB) [file 12879_2014_3996_MOESM1_ESM.docx]

**­Supplemental information**

**The distribution of incubation and relapse times in experimental human infections with the malaria parasite *Plasmodium vivax***

Andrew A. Lover, Xiahong Zhao, Zheng Gao, Richard J. Coker, and Alex R. Cook

Table of Contents

Section I. Posterior values for best-fit distributions 2

Section II. Sensitivity analysis 9

A. Tables 9

B. Figures- aggregate data sensitivity analysis 11

C. Figures- subregion specific sensitivity analysis 16

Section II. Kaplan-Meier plots (incubation data) 19

Section III. Kaplan-Meier plots and censoring (relapse data) 20

Section IV. Parameterization of distributions 23

## Section I. Posterior values for best-fit distributions

| Temperate  Old World | Shape  (95% CI) | Scale  (95% CI) | Shift  (95% CI) |
| --- | --- | --- | --- |
| Posterior mean | 1.7×10^-01^  (1.2×10^-01^, 2.3×10^-01^) | 2.8 (2.6, 3.0) | 2.5 (0.1, 6.2) |
| Covariance | Shape | Scale | Shift |
| Shape | 8.06×10^-04^ | -2.17×10^-03^ | 3.42×10^-02^ |
| Scale | -2.17×10^-03^ | 1.20×10^-02^ | -1.72×10^-01^ |
| Shift | 3.42×10^-02^ | -1.72×10^-01^ | 2.80 |

Table S1a. Model parameters with covariance matrix for shifted log-logistic distributions fitted to incubation times for the Temperate Old World quadrant (region-specific best-fit distribution).

| Temperate  Old World | Shape  (95% CI) | Scale  (95% CI) |
| --- | --- | --- |
| Posterior mean | 1.4×10^-01^  (1.2×10^-01^, 1.8×10^-01^) | 3.0 (2.9, 3.0) |
| Covariance | Shape | Scale |
| Shape | 2.79×10^-04^ | -2.07×10^-05^ |
| Scale | -2.07×10^-05^ | 1.06×10^-03^ |

Table S1b. Model parameters with covariance matrix of log-logistic distributions fitted to incubation times for the Temperate Old World quadrant (aggregate data best-fit distribution).

| Temperate  New World | Shape  (95% CI) | Scale  (95% CI) | Shift  (95% CI) |
| --- | --- | --- | --- |
| Posterior mean | 4.5  (3.1, 5.9) | 12.0  (8.9, 15.0) | 3.0  (0.2, 5.9) |
| Covariance | Shape | Scale | Shift |
| Shape | 0.56 | 1.16 | -1.10 |
| Scale | 1.16 | 2.82 | -2.68 |
| Shift | -1.10 | -2.68 | 2.60 |

Table S2a. Model parameters with covariance matrix of shifted Weibull distributions fitted to incubation times in the Temperate New World quadrant (region-specific best-fit distribution).

| Temperate  New World | Shape  (95% CI) | Scale  (95% CI) | Shift  (95% CI) |
| --- | --- | --- | --- |
| Posterior mean | 1.4×10^-01^  (1.1×10^-01^, 1.8×10^-01^) | 2.5 (2.3, 2.6) | 1.2 (0.3, 3.6) |
| Covariance | Shape | Scale | Shift |
| Shape | 2.66×10^-04^ | -1.09×10^-03^ | 1.29×10^-02^ |
| Scale | -1.09×10^-03^ | 7.14×10^-03^ | -8.06×10^-02^ |
| Shift | 1.29×10^-02^ | -8.06×10^-02^ | 9.65×10^-01^ |

Table S2b. Model Parameters with covariance matrix of shifted log-logistic distributions fitted to incubation times in the Temperate New World quadrant (aggregate data best-fit distribution).

| Tropical  Old World | Shape  (95% CI) | Rate  (95% CI) | Shift  (95% CI) |
| --- | --- | --- | --- |
| Posterior mean | 1.6×10^-01^  (1.0×10^-01^, 2.4×10^-01^) | 2.1  (1.7, 2.4) | 3.9  (0.4, 6.6) |
| Covariance | Shape | Rate | Shift |
| Shape | 1.33×10^-03^ | -7.26×10^-03^ | 5.59×10^-02^ |
| Rate | -7.26×10^-03^ | 4.40×10^-02^ | -3.43×10^-01^ |
| Shift | 5.59×10^-02^ | -3.43×10^-01^ | 2.72 |

Table S3. Model parameters with covariance matrix of shifted log-logistic distributions fitted to incubation times in the Tropical Old World quadrant (aggregate & region-specific data best-fit distribution).

| Tropical  New World | Shape  (95% CI) | Rate  (95% CI) | Shift  (95% CI) |
| --- | --- | --- | --- |
| Posterior mean | 1.8×10^-01^  (9.0×10^-02^, 3.4×10^-01^) | 2.1 (1.4, 2.6) | 4.8 (0.2, 8.7) |
| Covariance | Shape | Scale | Shift |
| Shape | 4.88×10^-03^ | -2.00×10^-02^ | 1.46×10^-01^ |
| Rate | -2.00×10^-02^ | 1.03×10^-01^ | -7.57×10^-01^ |
| Shift | 1.46×10^-01^ | -7.57×10^-01^ | 5.91 |

Table S4a. Model parameters with covariance matrix of shifted log-logistic distributions fitted to incubation times for the Tropical New World quadrant (aggregate data best-fit distribution).

| Tropical  New World | Shape  (95% CI) | Rate  (95% CI) | Shift  (95% CI) |
| --- | --- | --- | --- |
| Posterior mean | 4.9×10^-01^  (3.7×10^-02^, 6.2×10^-01^) | 2.6×10^-03^  (3.3×10^-04^, 8.3×10^-03^) | 2.6 (0.6, 4.6) |
| Covariance | Shape | Rate | Shift |
| Shape | 4.06×10^-03^ | -9.53×10^-05^ | 9.50×10^-04^ |
| Rate | -9.53×10^-05^ | 5.10×10^-06^ | 1.24×10^-03^ |
| Shift | 9.50×10^-04^ | 1.24×10^-03^ | 1.04 |

Table S4b. Model parameters with covariance matrix of shifted Gompertz distributions fitted to incubation times for the Tropical New World quadrant (region-specific best-fit distribution).

| Overall | Shape (95% CI) | Scale (95% CI) | Shift (95% CI) |
| --- | --- | --- | --- |
| Posterior mean | 0.3 (0.2,0.3) | 2.0 (1.8, 2.1) | 6.1 (5.1, 6.8) |
| Covariance | Shape | Scale | Shift |
| Shape | 2.15×10^-03^ | -7.11×10^-02^ | -1.28×10^-02^ |
| Scale | -7.11×10^-03^ | 3.14×10^-04^ | 4.66×10^-03^ |
| Shift | -1.28×10^-02^ | 4.66×10^-03^ | 8.28×10^-01^ |

Table S4. Model parameters with covariance matrix of shifted log-logistic distribution for aggregate incubation period data (all regions).

| Temperate  Old World | Shape_1_  (95% CI) | Scale_1_  (95% CI) | Shape_2_  (95% CI) | Scale_2_  (95% CI) | P  (95% CI) |
| --- | --- | --- | --- | --- | --- |
| posterior mean | 7.3×10^-01^ | 3.7 | 3.3×10^-01^ | 7.8×10^-01^ | 0.3 |
|  | (6.2×10^-01^,  8.5×10^-01^) | (3.7, 3.8) | (3.0×10^-01^,  3.8×10^-01^) | (7.2×10^-01^,  8.5×10^-01^) | (0.2, 0.3) |
| Covariance | Shape_1_ | Scale_1_ | Shape_2_ | Scale_2_ | P |
| Shape_1_ | 6.80×10^-06^ | -3.63×10^-08^ | 6.11×10^-06^ | -2.21×10^-05^ | 1.90×10^-05^ |
| Scale_1_ | -3.63×10^-08^ | 4.23×10^-05^ | -4.31×10^-05^ | 1.75×10^-05^ | 2.35×10^-05^ |
| Shape_2_ | 6.11×10^-06^ | -4.31×10^-05^ | 2.48×10^-04^ | -2.07×10^-04^ | -7.38×10^-05^ |
| Scale_2_ | -2.21×10^-05^ | 1.75×10^-05^ | -2.07×10^-04^ | 3.08×10^-04^ | -3.27×10^-05^ |
| P | 1.90×10^-05^ | 2.35×10^-05^ | -7.38×10^-05^ | -3.27×10^-05^ | 2.16×10^-04^ |

Table S6. Model parameters with covariance matrix of mixture log-logistic distributions fitted to long-latent incubation times (aggregate & region-specific best-fit distribution).

| Temperate  Old World | Shape_1_  (95% CI) | Rate_1_  (95% CI) | Shape_2_  (95% CI) | Rate_2_  (95% CI) | P  (95% CI) |
| --- | --- | --- | --- | --- | --- |
| posterior mean | 5.7×10^-02^ | 3.4×10^-03^ | 1.1×10^-02^ | 5.9×10^-04^ | 2.3×10^-01^ |
|  | (2.6×10^-02^,  1.0×10^-01^) | (3.3×10^-04^,  9.6×10^-03^) | (8.3×10^-03^,  1.4×10^-02^) | (2.7×10^-04^,  1.1×10^-03^) | (1.3×10^-01^, 3.2×10^-01^) |
| Covariance | Shape_1_ | Rate_1_ | Shape_2_ | Rate_2_ | P |
| Shape_1_ | 3.80×10^-04^ | -3.87×10^-05^ | -1.68×10^-06^ | 2.50×10^-07^ | -1.48×10^-04^ |
| Rate_1_ | -3.87×10^-05^ | 5.99×10^-06^ | 4.80×10^-08^ | 1.32×10^-09^ | -3.53×10^-06^ |
| Shape_2_ | -1.68×10^-06^ | 4.80×10^-08^ | 1.76×10^-06^ | -2.55×10^-07^ | 2.33×10^-05^ |
| Rate_2_ | 2.50×10^-07^ | 1.32×10^-09^ | -2.55×10^-07^ | 4.42×10^-08^ | -4.01×10^-06^ |
| P | -1.48×10^-04^ | -3.53×10^-06^ | 2.33×10^-05^ | -4.01×10^-06^ | 2.49×10^-03^ |

Table S7. Model parameters with covariance matrix for mixture Gompertz distribution fitted to relapse times for the Temperate Old World quadrant (aggregate & region-specific data best-fit distribution).

| Temperate  New World | Shape_1_  (95% CI) | Rate_1_  (95% CI) | Shape_2_  (95% CI) | Rate_2_  (95% CI) | P  (95% CI) |
| --- | --- | --- | --- | --- | --- |
| posterior mean | 4.7×10^-02^ | 2.9×10^-02^ | 3.0×10^-02^ | 1.3×10^-05^ | 2.1×10^-01^ |
|  | (1.4×10^-03^,  1.1×10^-01^) | (6.9×10^-03^,  6.9×10^-02^) | (2.2×10^-02^,  4.1×10^-02^) | (4.4×10^-07^,  5.6×10^-05^) | (1.0×10^-01^, 3.4×10^-01^) |
| Covariance | Shape_1_ | Rate_1_ | Shape_2_ | Rate_2_ | P |
| Shape_1_ | 9.98×10^-04^ | -3.13×10^-04^ | -3.31×10^-05^ | 7.13×10^-08^ | -2.42×10^-04^ |
| Rate_1_ | -3.13×10^-04^ | 2.54×10^-04^ | 1.13×10^-06^ | -4.34×10^-09^ | -1.90×10^-05^ |
| Shape_2_ | -3.31×10^-05^ | 1.13×10^-06^ | 2.20×10^-05^ | -5.61×10^-08^ | 3.68×10^-05^ |
| Rate_2_ | 7.13×10^-08^ | -4.34×10^-09^ | -5.61×10^-08^ | 2.34×10^-10^ | -8.79×10^-08^ |
| P | -2.42×10^-04^ | -1.90×10^-05^ | 3.68×10^-05^ | -8.79×10^-08^ | 3.87×10^-03^ |

Table S8. Model parameters with covariance matrix for mixed Gompertz distribution fitted to relapse times for the Temperate New World quadrant (aggregate & region-specific best-fit distribution).

| Tropical  Old World | Shape_1_  (95% CI) | Rate_1_  (95% CI) | Shape_2_  (95% CI) | Rate_2_  (95% CI) | P  (95% CI) |
| --- | --- | --- | --- | --- | --- |
| posterior mean | 2.5×10^-01^ | 1.0×10^-03^ | 2.4×10^-03^ | 8.8×10^-03^ | 6.4×10^-01^ |
|  | (1.4×10^-01^,  3.7×10^-01^) | (4.1×10^-05^,  4.5×10^-03^) | (9.5×10^-05^,  6.9×10^-03^) | (3.0×10^-03^,  1.7×10^-02^) | (4.1×10^-01^, 8.3×10^-01^) |
| Covariance | Shape_1_ | Rate_1_ | Shape_2_ | Rate_2_ | P |
| Shape_1_ | 3.41×10^-03^ | -5.61×10^-05^ | -2.20×10^-06^ | 1.83×10^-05^ | -1.05×10^-03^ |
| Rate_1_ | -5.61×10^-05^ | 1.48×10^-06^ | -2.02×10^-10^ | -1.79×10^-07^ | 1.29×10^-05^ |
| Shape_2_ | -2.20×10^-06^ | -2.02×10^-10^ | 3.57×10^-06^ | -3.71×10^-06^ | 1.79×10^-05^ |
| Rate_2_ | 1.83×10^-05^ | -1.79×10^-07^ | -3.71×10^-06^ | 1.27×10^-05^ | -7.04×10^-05^ |
| P | -1.05×10^-03^ | 1.29×10^-05^ | 1.79×10^-05^ | -7.04×10^-05^ | 1.15×10^-02^ |

Table S9. Model parameters with covariance matrix for mixed Gompertz distribution fitted to relapse times for the Tropical Old World quadrant (aggregate & region-specific best-fit distribution).

| Tropical  New World | Shape_1_  (95% CI) | Rate_1_  (95% CI) | Shape_2_  (95% CI) | Rate_2_  (95% CI) | P  (95% CI) |
| --- | --- | --- | --- | --- | --- |
| posterior mean | 2.2×10^-02^ | 4.8×10^-04^ | 2.6 | 2.7 | 9.8×10^-01^ |
|  | (1.4×10^-02^,  3.0×10^-02^) | (9.5×10^-05^,  1.4×10^-03^) | (0.1, 7.2) | (0.1, 7.4) | (8.9×10^-01^, 1.0×10^-01^) |
| Covariance | Shape_1_ | Rate_1_ | Shape_2_ | Rate_2_ | P |
| Shape_1_ | 1.82×10^-05^ | -1.29×10^-06^ | 7.36×10^-05^ | 7.01×10^-04^ | -4.08×10^-06^ |
| Rate_1_ | -1.29×10^-06^ | 1.18×10^-07^ | -1.29×10^-05^ | -3.20×10^-05^ | 4.26×10^-07^ |
| Shape_2_ | 7.36×10^-05^ | -1.29×10^-05^ | 3.81 | -3.66×10^-02^ | -2.54×10^-04^ |
| Rate_2_ | 7.01×10^-04^ | -3.20 ×10^-05^ | -3.66×10^-02^ | 3.75 | 4.84×10^-04^ |
| P | -4.08×10^-06^ | 4.26×10^-07^ | -2.54×10^-04^ | 4.84×10^-04^ | 9.89×10^-04^ |

Table S10a. Model parameters with covariance matrix for mixed Gompertz distributions fitted to relapse times for the Tropical New World quadrant (aggregate data best-fit distribution).

| Tropical  New World | Shape  (95% CI) | Rate  (95% CI) | Shift  (95% CI) |
| --- | --- | --- | --- |
| posterior mean | 1.8×10^-02^ | 3.5×10^-03^ | 75.8 |
|  | (7.0×10^-03^,  2.8×10^-02^) | (1.1×10^-03^,  8.7×10^-03^) | (75.7, 75.9) |
| Covariance | Shape_1_ | Rate_1_ | Shape_2_ |
| Shape | 2.96×10^-05^ | -9.26×10^-06^ | 7.44×10^-06^ |
| Rate | -9.26×10^-06^ | 3.77×10^-06^ | -3.41×10^-06^ |
| Shift | 7.44×10^-06^ | -3.41×10^-06^ | 1.46×10^-03^ |

Table S10b. Model parameters with covariance matrix for shifted Gompertz distributions fitted to relapse times for the Tropical New World quadrant (region-specific best-fit distribution).

| Overall | Shape_1_ (95% CI) | Rate_1_  (95% CI) | Shape_2_  (95% CI) | Rate_2_  (95% CI) | P  (95% CI) |
| --- | --- | --- | --- | --- | --- |
| posterior mean | 7.4×10^-04^ | 8.2×10^-03^ | 2.7×10^-02^ | 1.5×10^-05^ | 5.5×10^-01^ |
|  | (6.2×10^-05^,  1.5×10^-03^) | (6.9×10^-03^,  9.6×10^-03^) | (2.4×10^-02^,  3.0×10^-02^) | (6.3×10^-06^,  2.9×10^-05^) | (4.8×10^-01^, 6.1×10^-01^) |
| Covariance | Shape_1_ | Rate_1_ | Shape_2_ | Rate_2_ | P |
| Shape_1_ | 1.44×10^-07^ | -1.58×10^-07^ | 6.18×10^-08^ | -1.94×10^-10^ | 3.37×10^-06^ |
| Rate_1_ | -1.58×10^-07^ | 5.36×10^-07^ | -2.39×10^-07^ | 8.21×10^-10^ | -1.17×10^-05^ |
| Shape_2_ | 6.18×10^-08^ | -2.39×10^-07^ | 2.51×10^-06^ | -9.63×10^-09^ | 1.65×10^-05^ |
| Rate_2_ | -1.94×10^-10^ | 8.21×10^-10^ | -9.63×10^-09^ | 4.04×10^-11^ | -6.70×10^-08^ |
| P | 3.37×10^-06^ | -1.17×10^-05^ | 1.65×10^-05^ | -6.70×10^-08^ | 1.15×10^-03^ |

Table S11. Model parameters with covariance matrix of mixed Gompertz for aggregate relapse period data (all regions).

## Section II. Sensitivity analysis

### A. Tables

|  | **DIC difference** | | | | |
| --- | --- | --- | --- | --- | --- |
|  | Original Data | Variance =0.01 | Variance =0.05 | Variance =0.1 | Variance =0.2 |
| **Incubation times** | Ref. | 1.5 | 17.6 | -8.4 | 75.6 |
| **Long-latency times** | Ref. | -11.5 | 14.8 | 72.1 | 355.3 |
| **Relapse times** | Ref. | -2.2 | 12.1 | 45.1 | 124.3 |

Table S12. DIC values for pseudodata with added variance ‘noise’ to examine sensitivity of best-fit distributions to the model selection procedure (aggregate data best-fit distribution).

| **Incubation Times** | **DIC difference** | | | | | |
| --- | --- | --- | --- | --- | --- | --- |
|  | Original Data | Variance =0.01 | Variance =0.05 | Variance =0.1 | Variance =0.2 | |
| New World Temperate | Ref. | -1.8 | 2.5 | 32.3 | 108.2 | |
| New World Tropical | Ref. | 0.7 | 7.5 | 18.3 | 29.8 | |
| Old World Temperate | Ref. | -0.2 | -0.6 | 12.1 | 34.0 | |
| Old World Tropical | Ref. | 1.0 | 18.8 | 75.0 | 103.8 | |
|  | | | | | |  |
| **Relapse Times** |  | | | | | |
| New World Temperate | Ref. | 2.2 | 17.1 | 22.3 | 39.0 | |
| New World Tropical | Ref. | -0.4 | -0.1 | -0.7 | 76.9 | |
| Old World Temperate | Ref. | 7.4 | 133.3 | 252.7 | 452.9 | |
| Old World Tropical | Ref. | 2.6 | 13.0 | 11.3 | 19.8 | |

Table S13. DIC value for pseudodata with added variance ‘noise’ to examine sensitivity best-fit distributions to the model selection procedure (region-specific best-fit distribution).

| **Incubation Times** | **DIC difference** | | | | | |
| --- | --- | --- | --- | --- | --- | --- |
|  | Original Data | Variance =0.01 | Variance =0.05 | Variance =0.1 | Variance =0.2 | |
| New World Temperate | Ref. | -1.5 | -6.3 | -6.4 | -136.3 | |
| New World Tropical | Ref. | 0.8 | -1.8 | 10.5 | -11.4 | |
| Old World Temperate | Ref. | -0.1 | -5.3 | -2.0 | -36.5 | |
|  | | | | | |  |
| **Relapse Times** |  | | | | | |
| New World Tropical | Ref. | 0.2 | -0.5 | 1.4 | 0.0 | |

Table S14. DIC values for pseudodata with added variance ‘noise’ to examine sensitivity of best-fit distributions to the model selection procedure (region-specific best-fit distribution).

### B. Figures- aggregate data sensitivity analysis

##
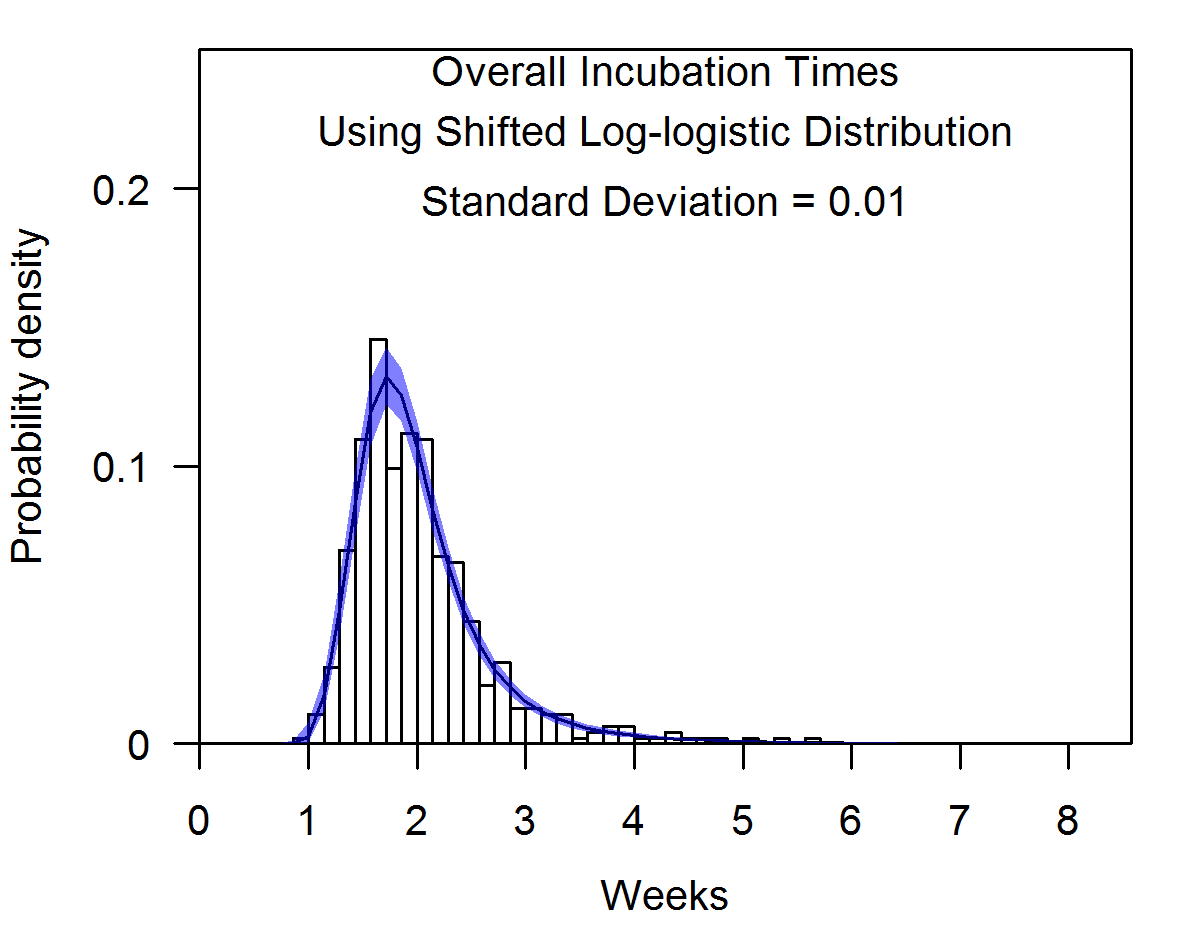


Figure S1. Sensitivity analysis of overall aggregate incubation period data (all regions) with 0.01 SD “noise” in dark blue.


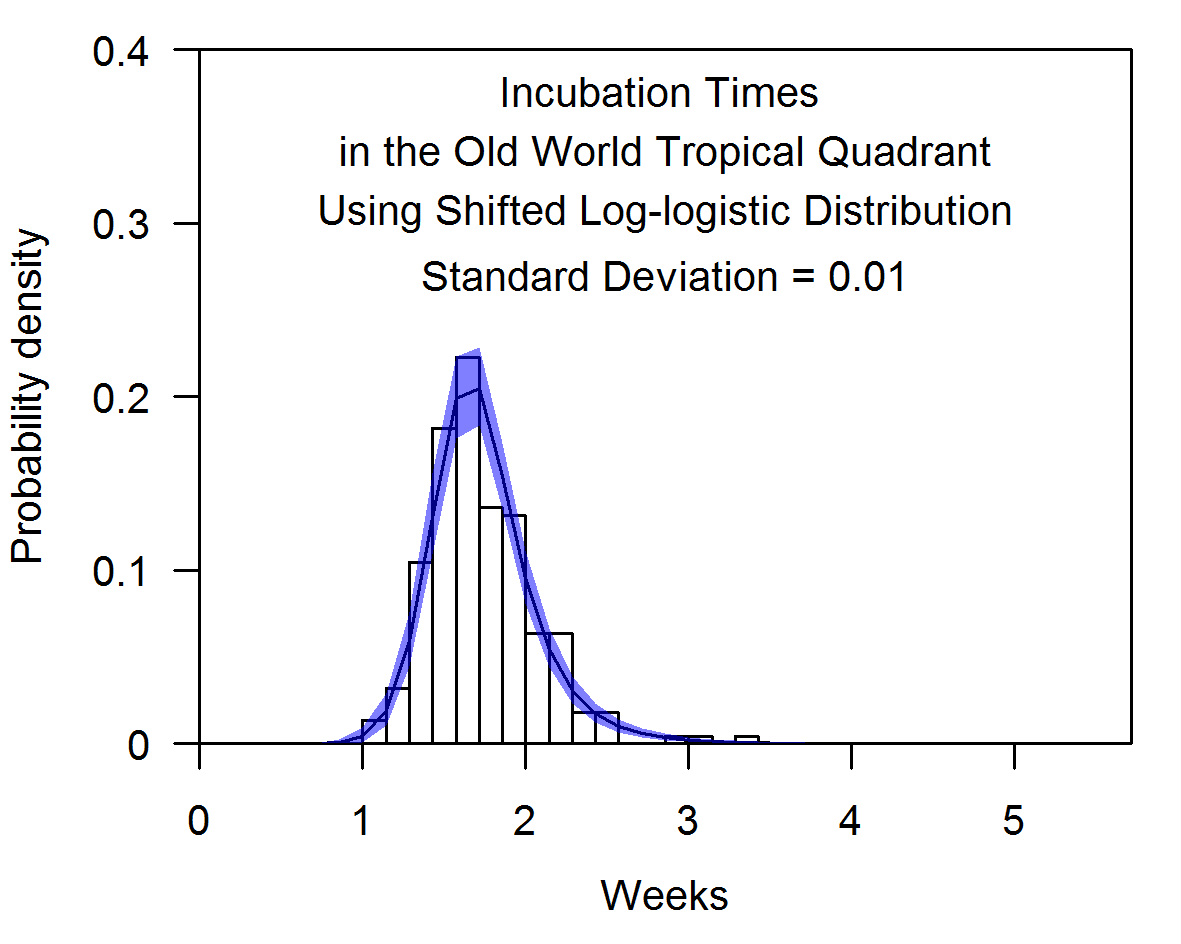


Figure S2. Sensitivity analysis of incubation period data (Old World Tropical region) with 0.01 SD “noise” in dark blue.

##
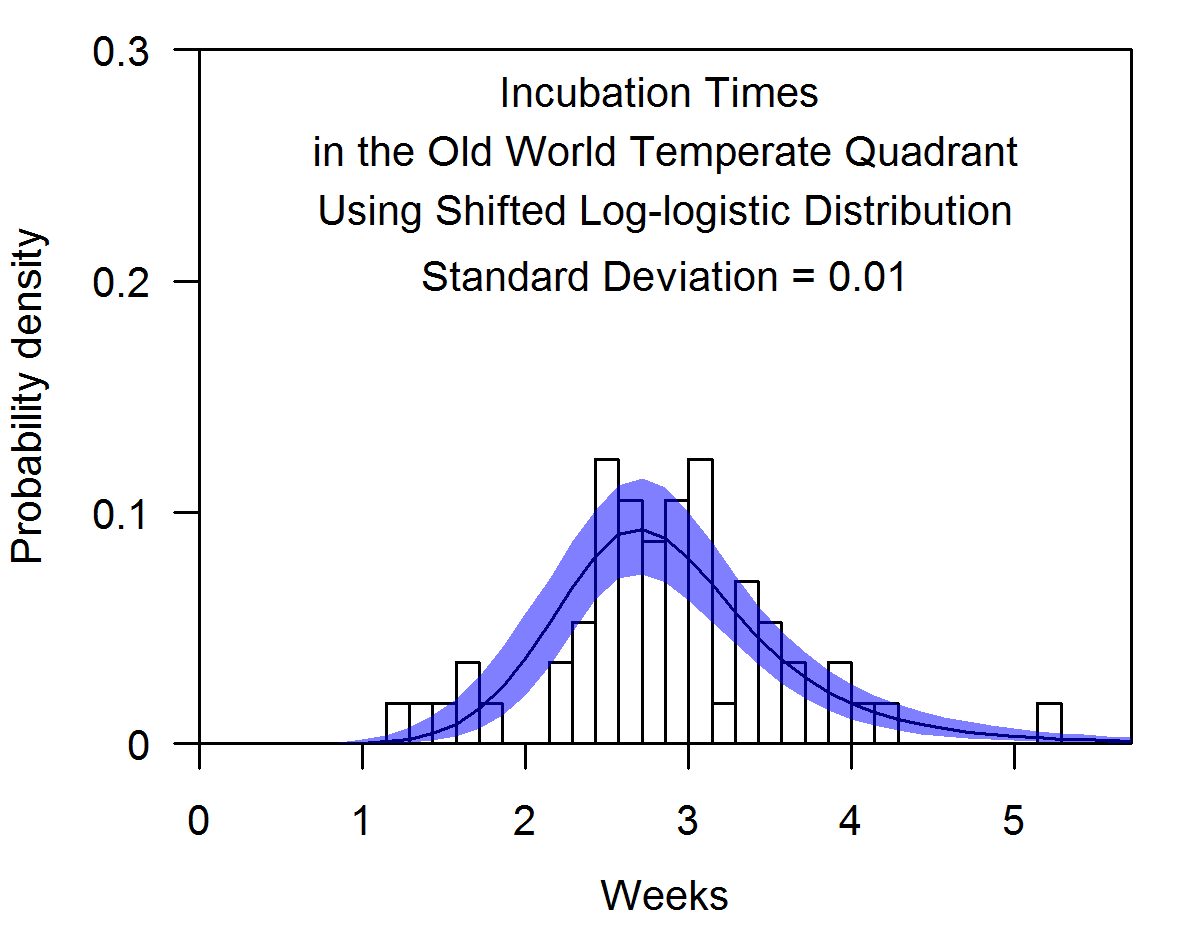


Figure S3. Sensitivity analysis of incubation period data (Old World Temperate region) with 0.01 SD “noise” in dark blue.


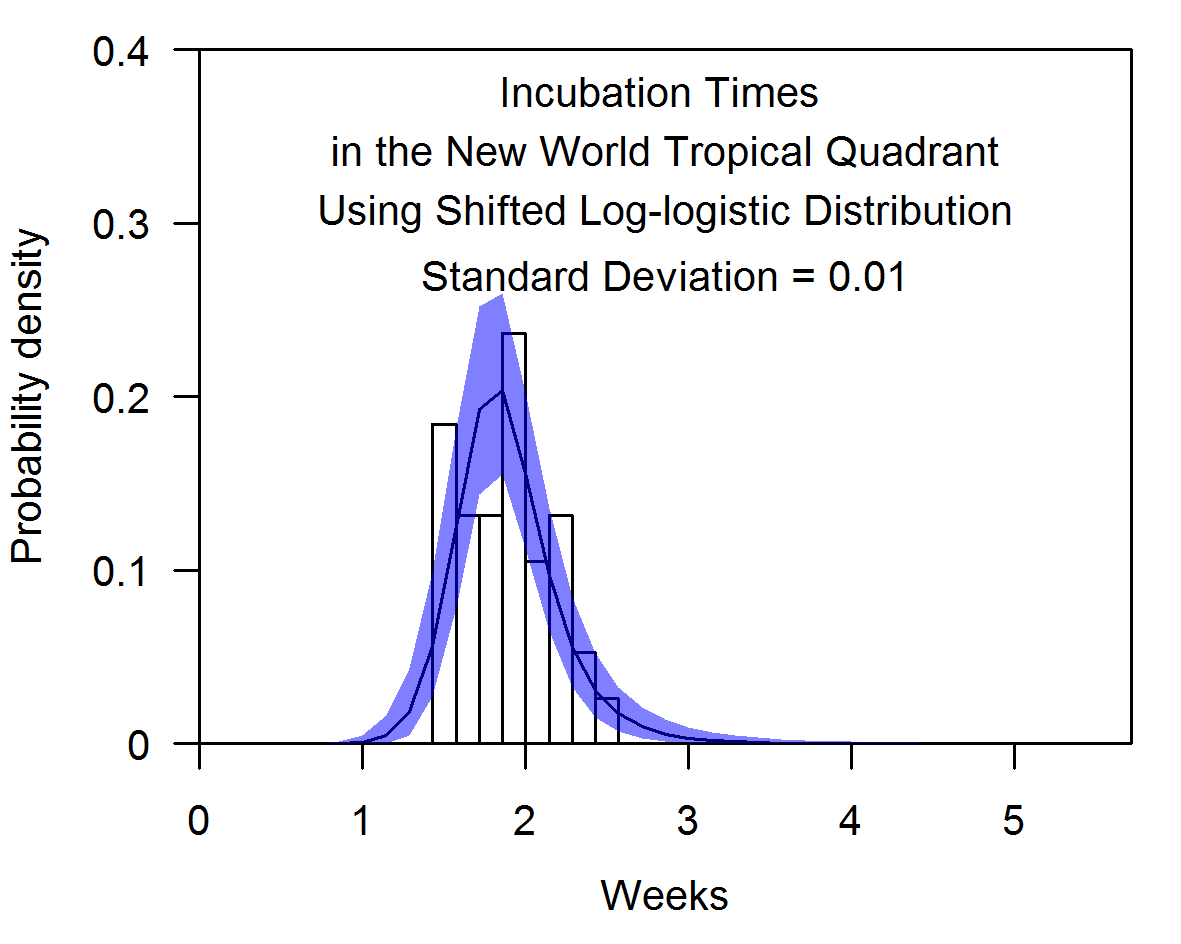


Figure S4. Sensitivity analysis of incubation period data (New World Tropical region) with 0.01 SD “noise” in dark blue.

##
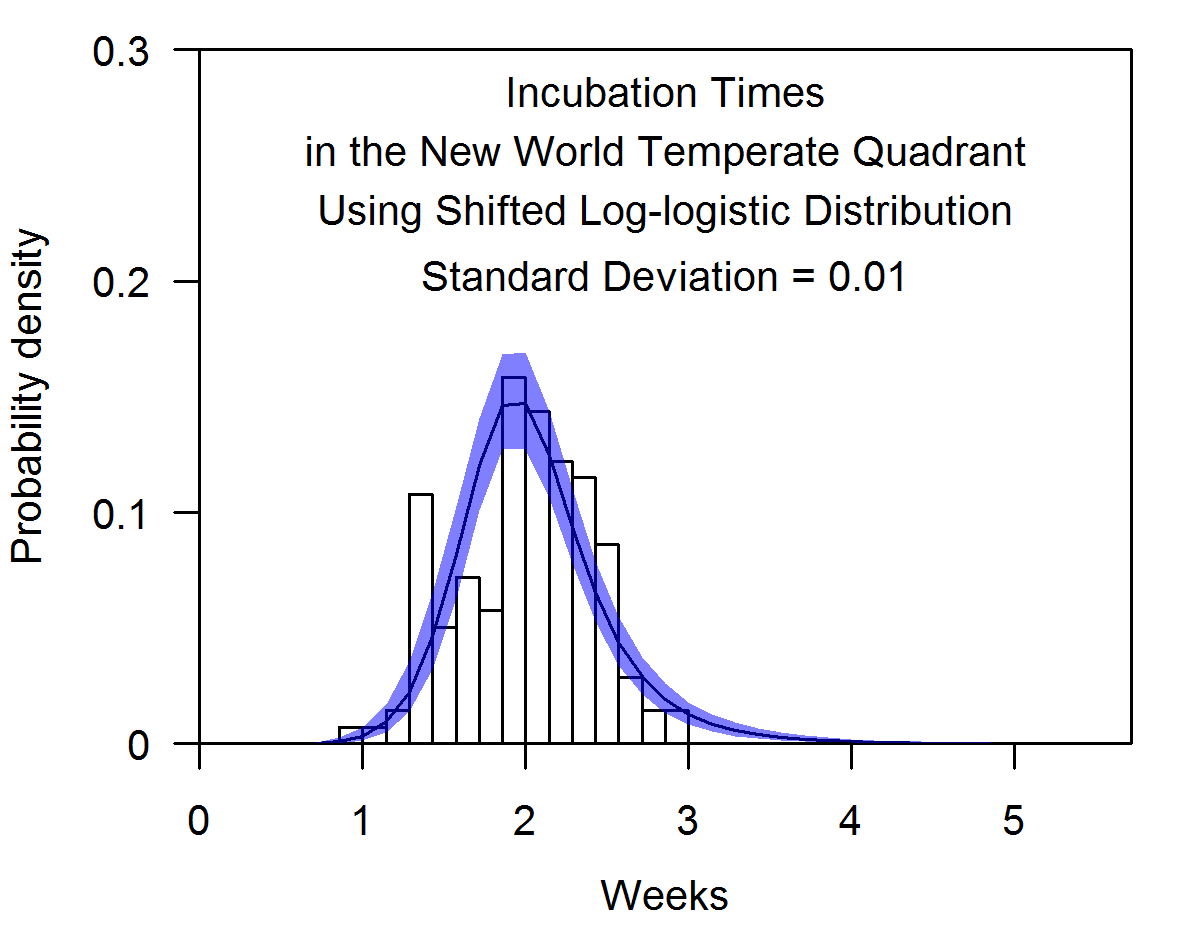


Figure S5. Sensitivity analysis of incubation period data (New World Temperate region) with 0.01 SD “noise” in dark blue.

##
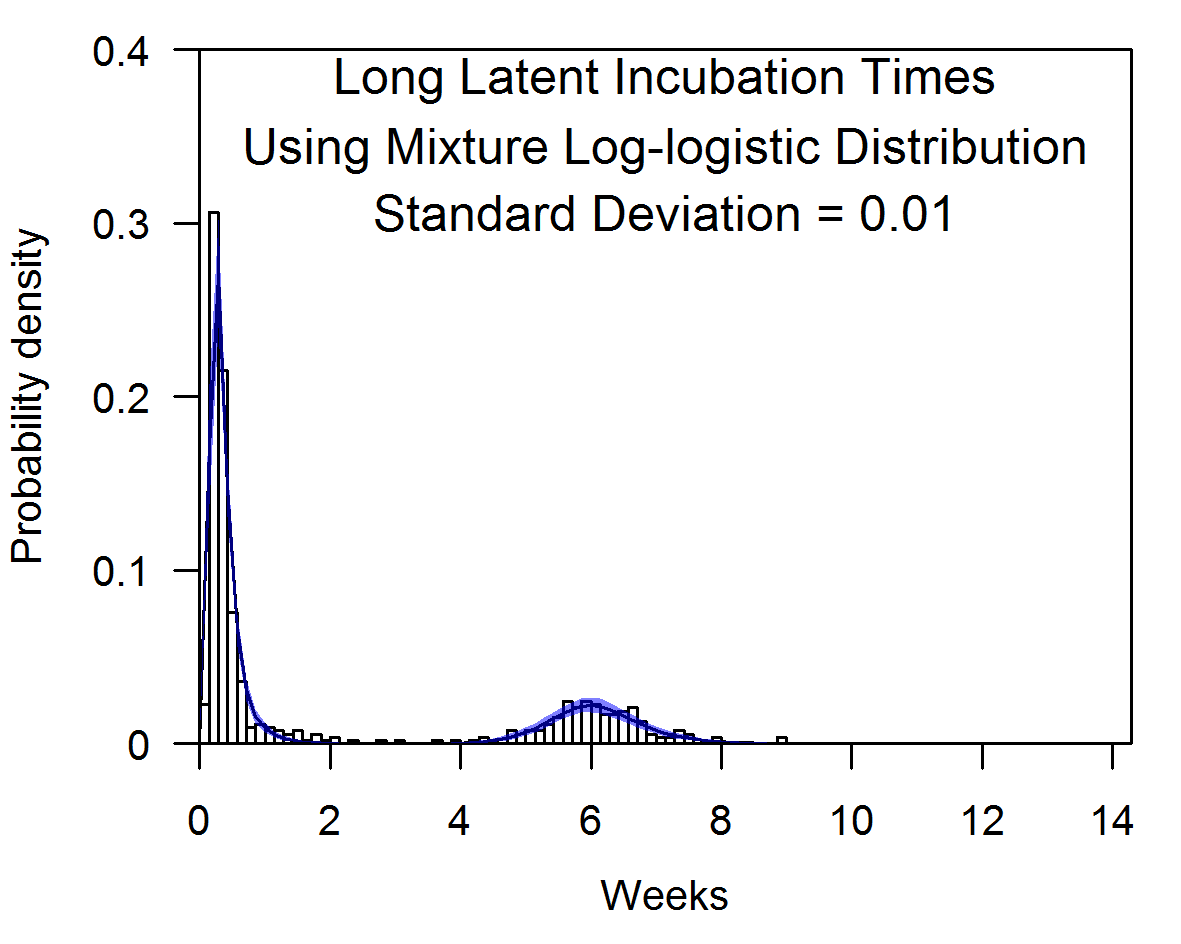


Figure S6. Sensitivity analysis of observational incubation period data with 0.01 SD “noise” in dark blue.

##
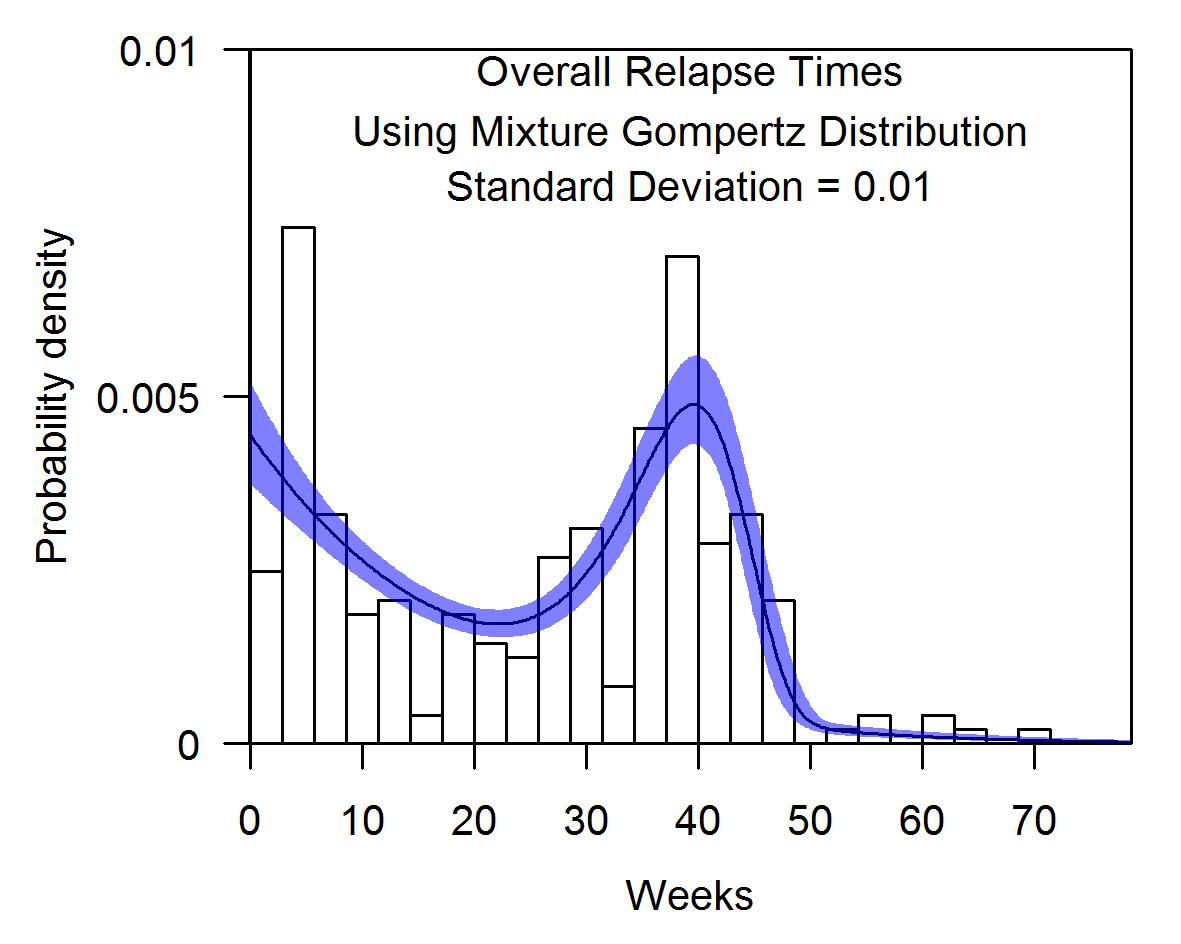


Figure S7. Sensitivity analysis of aggregate relapse period data (all regions) with 0.01 SD “noise” in dark blue.


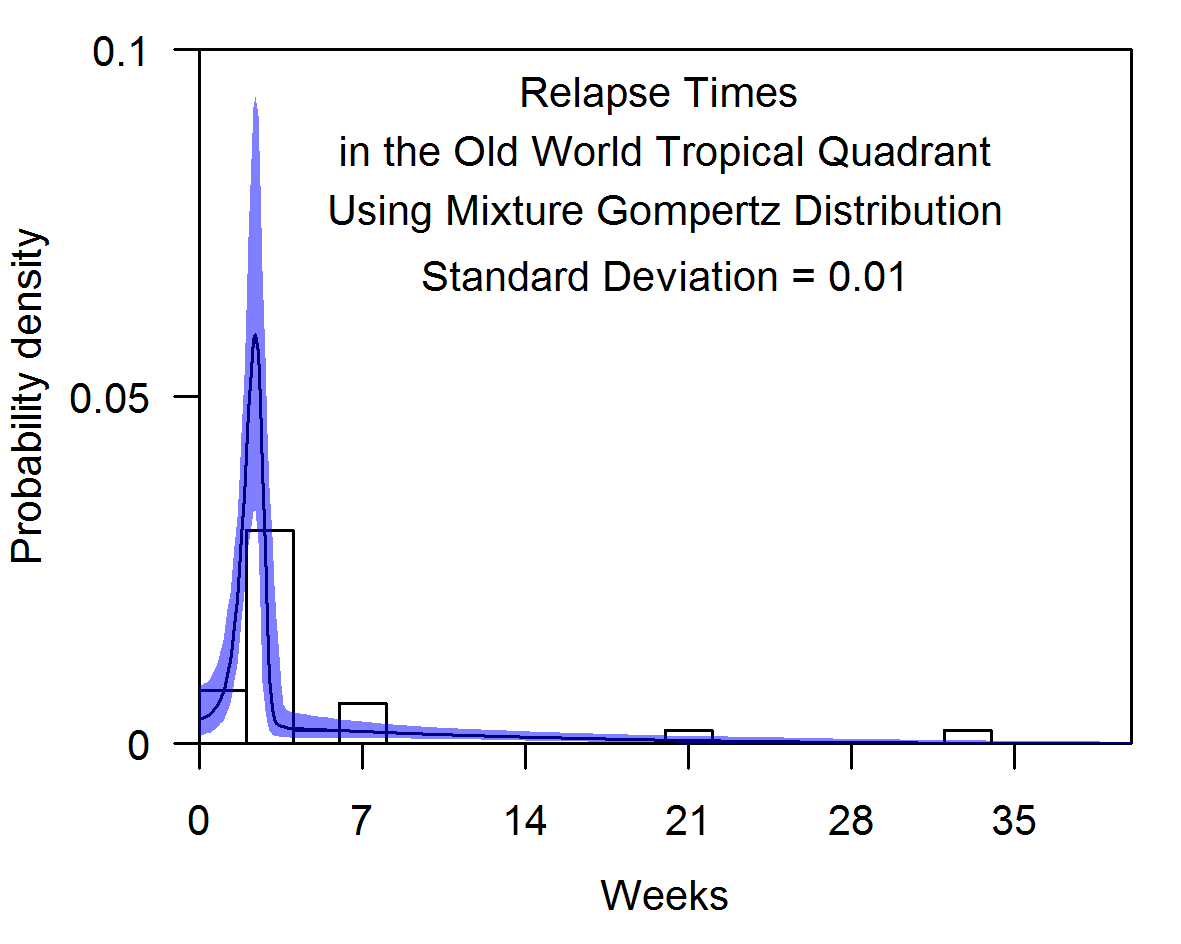


Figure S8. Sensitivity analysis of relapse period data (Old World Tropical region) with 0.01 SD “noise” in dark blue.


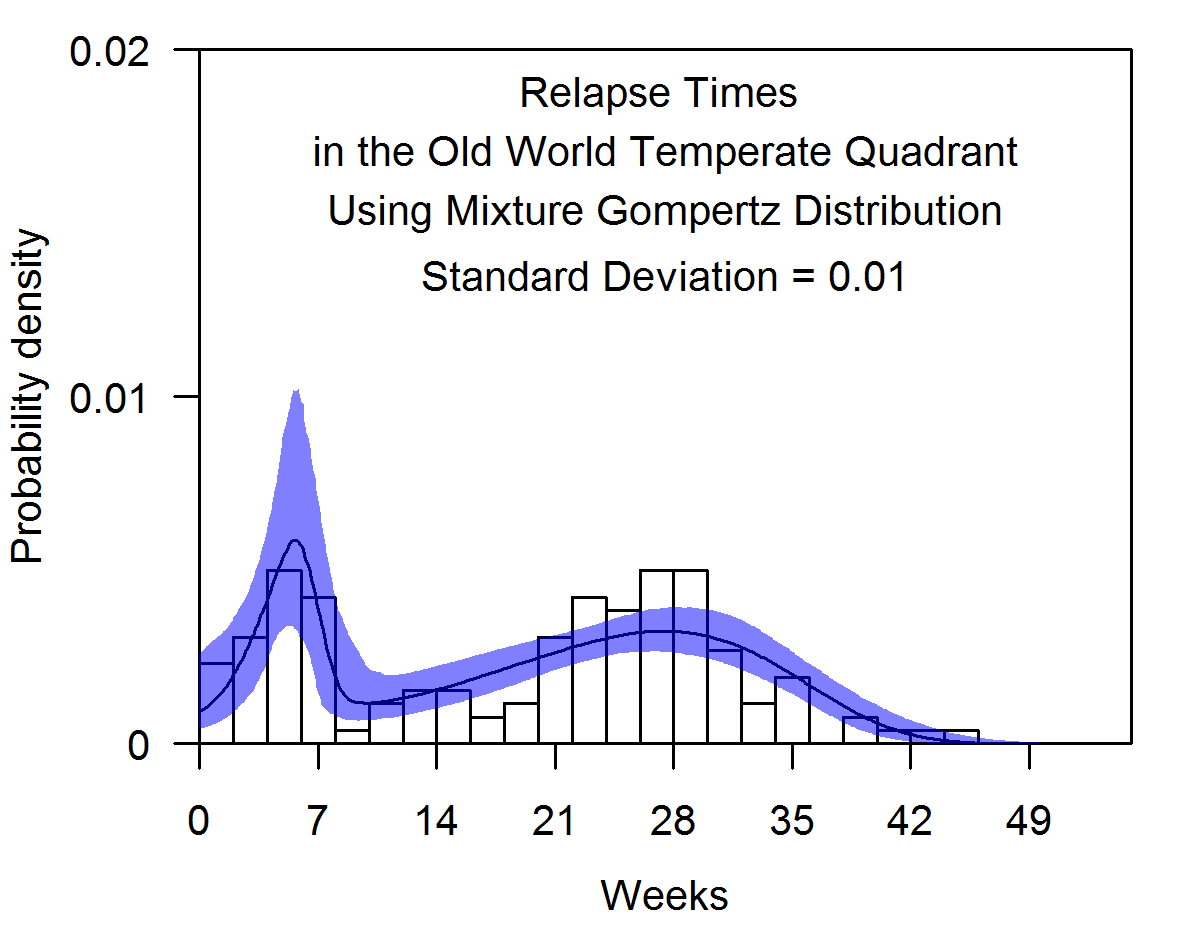


Figure S9. Sensitivity analysis of relapse period data (Old World Temperate region) with 0.01 SD “noise” in dark blue.


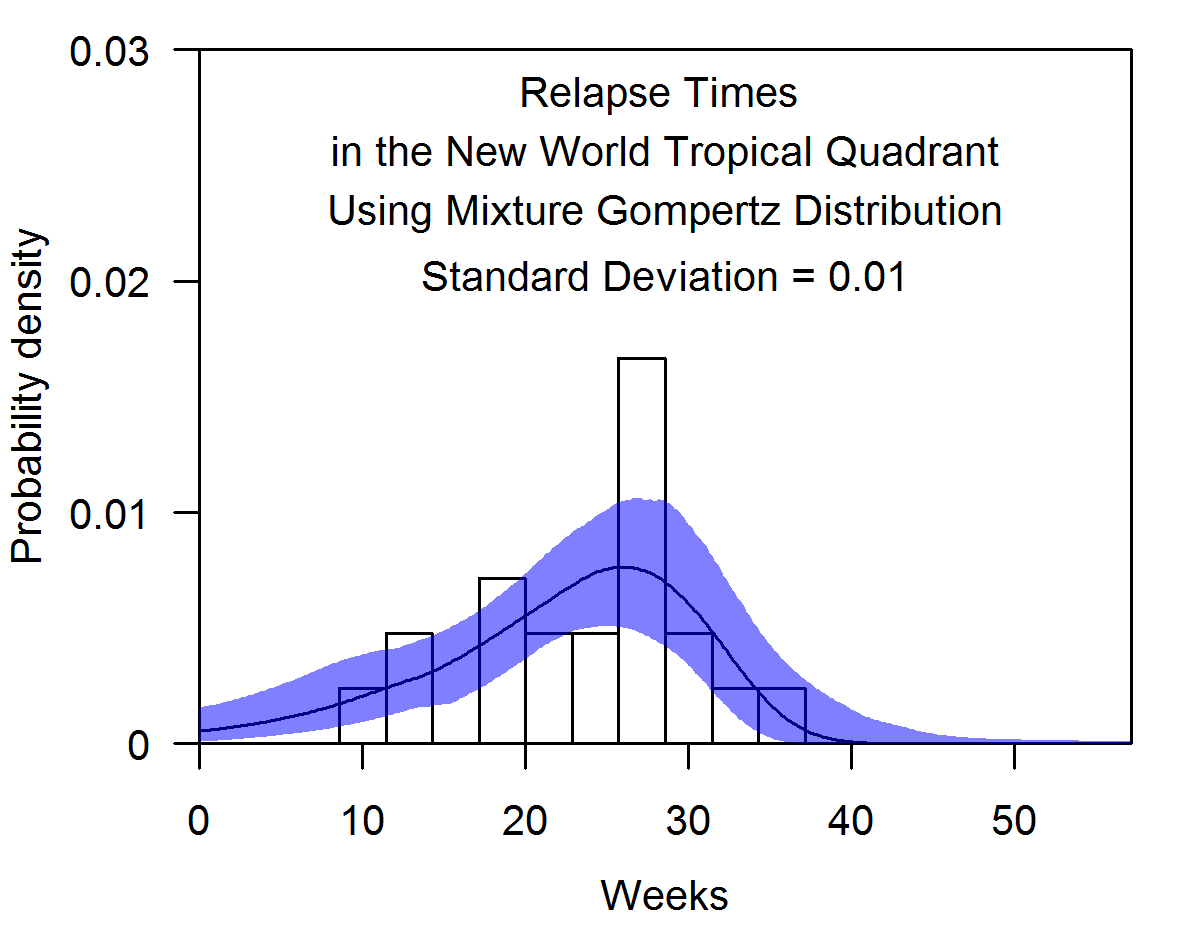


Figure S10. Sensitivity analysis of relapse period data (New World Tropical region) with 0.01 SD “noise” in dark blue.

##
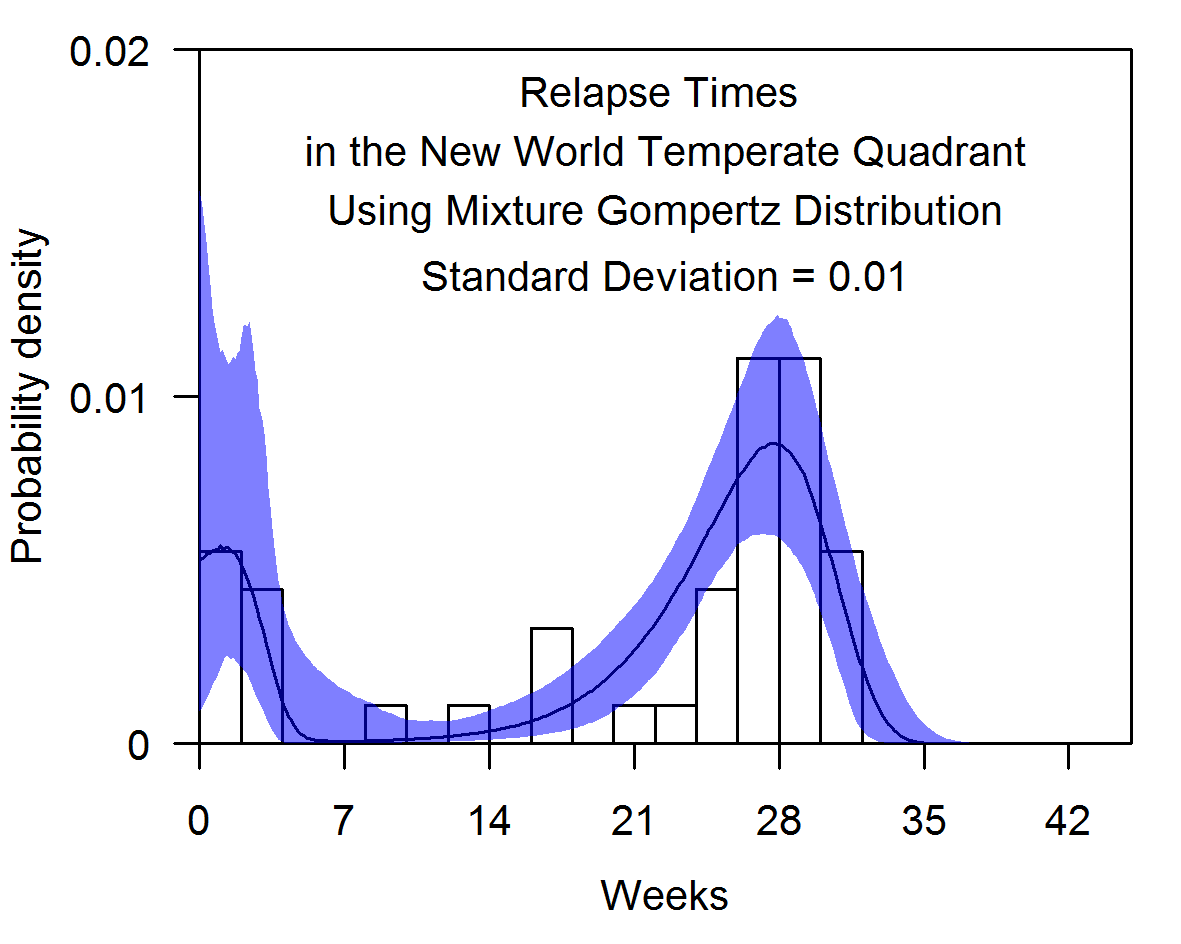


Figure S11. Sensitivity analysis of relapse period data (New World Temperate region) with 0.01 SD “noise” in dark blue.

### C. Figures- subregion specific sensitivity analysis

Note: these plots are only provided if the region-specific distribution differs from the best-fit from the aggregate data.


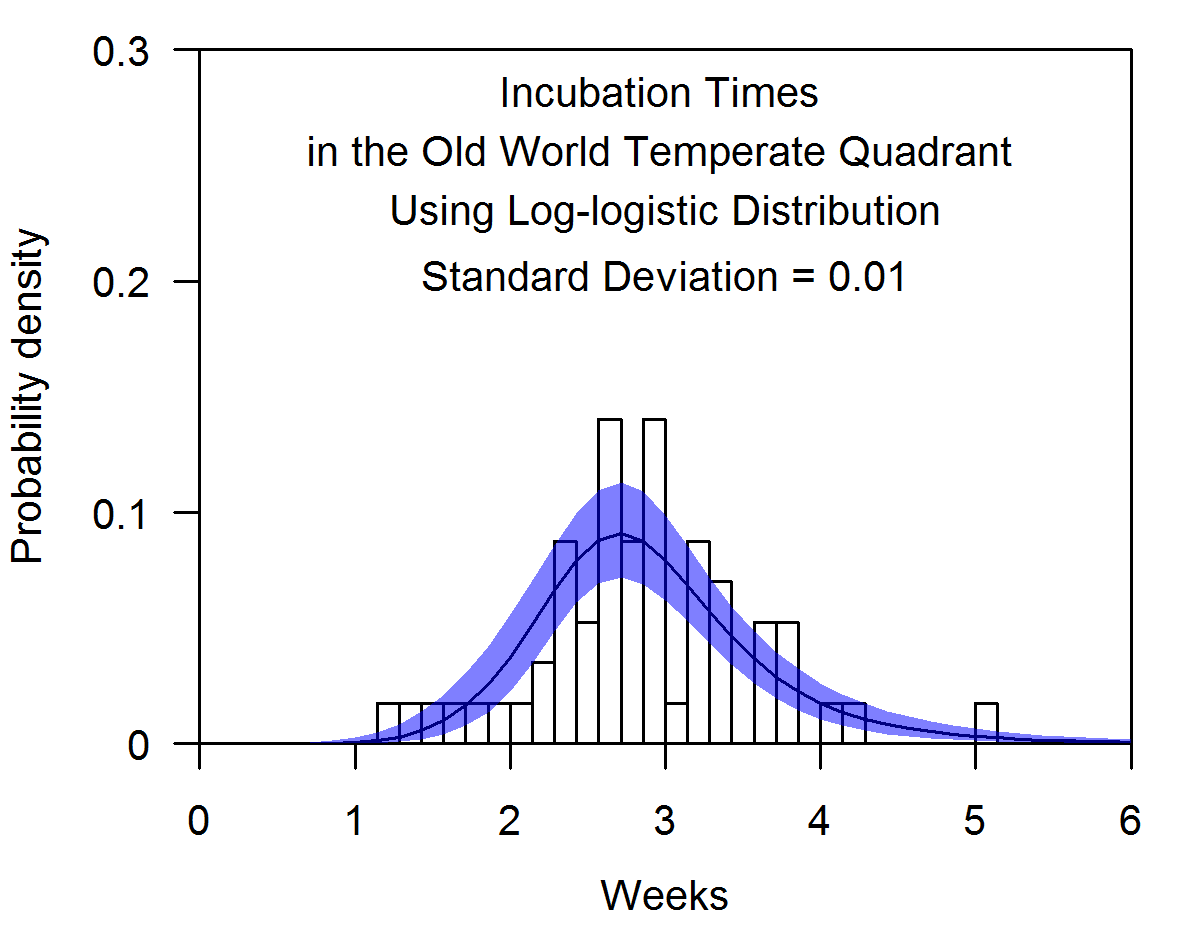


Figure S12. Sensitivity analysis of incubation period data (Old World Temperate region) with 0.01 SD “noise” in dark blue

##
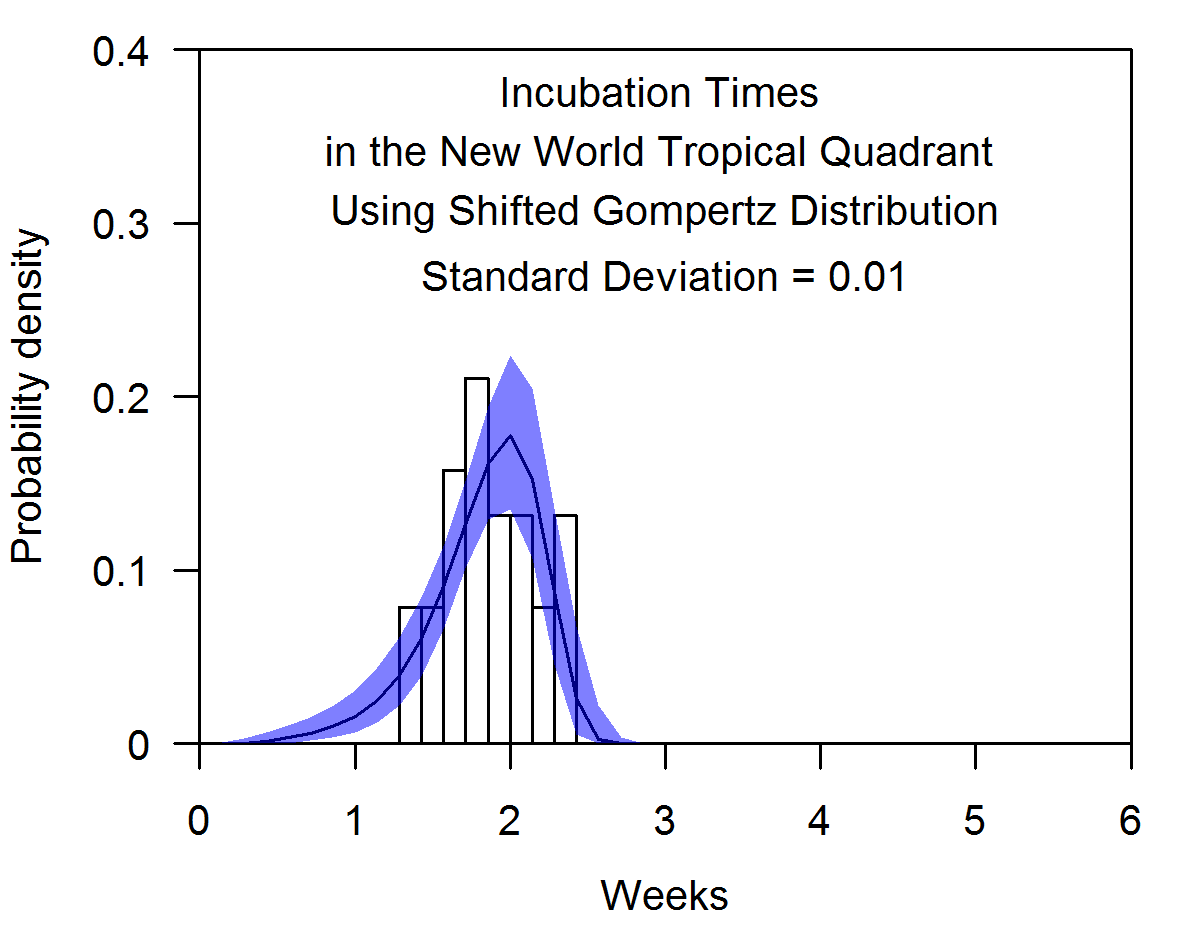


Figure S13. Sensitivity analysis of incubation period data (New World Tropical region) with 0.01 SD “noise” in dark blue

##
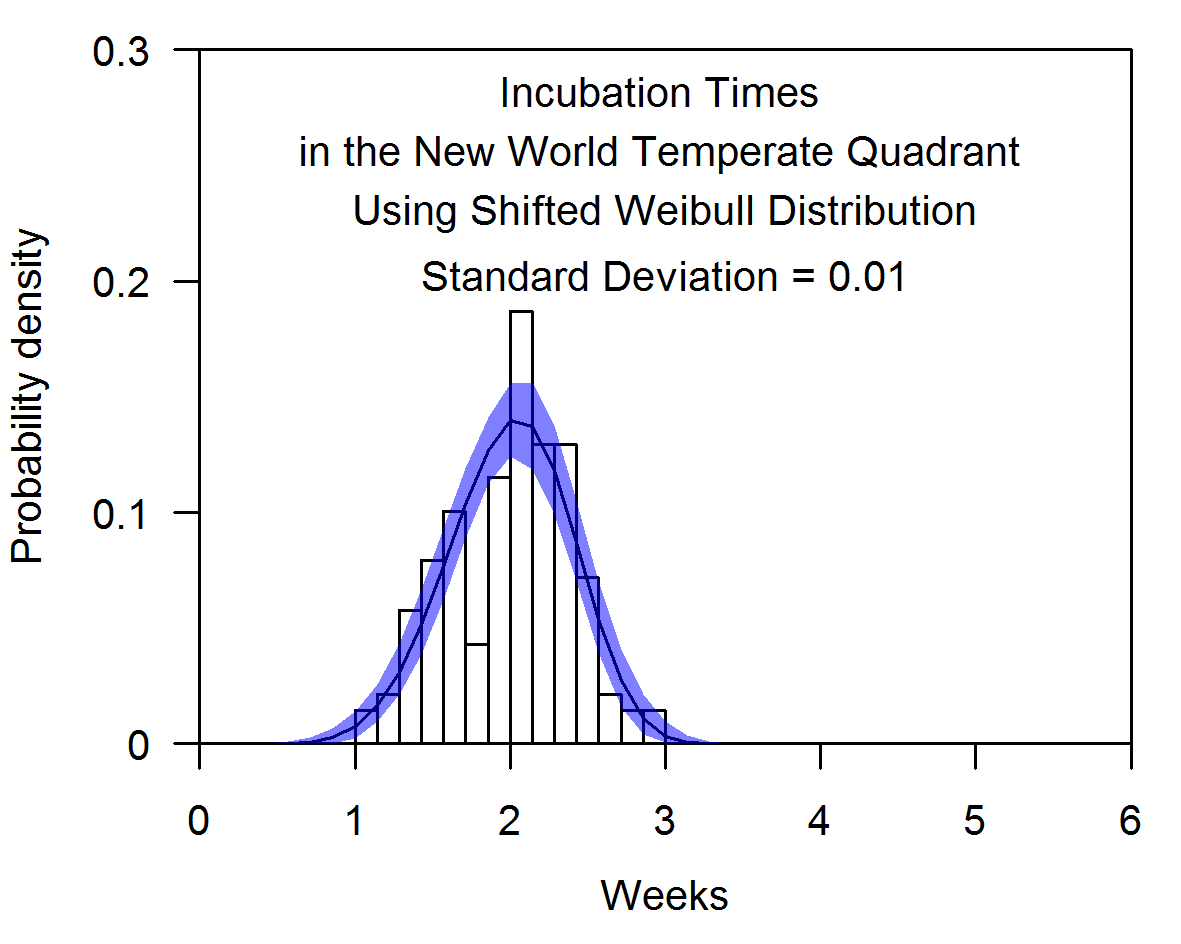


Figure S14. Sensitivity analysis of incubation period data (New World Temperate region) with 0.01 SD “noise” in dark blue

##
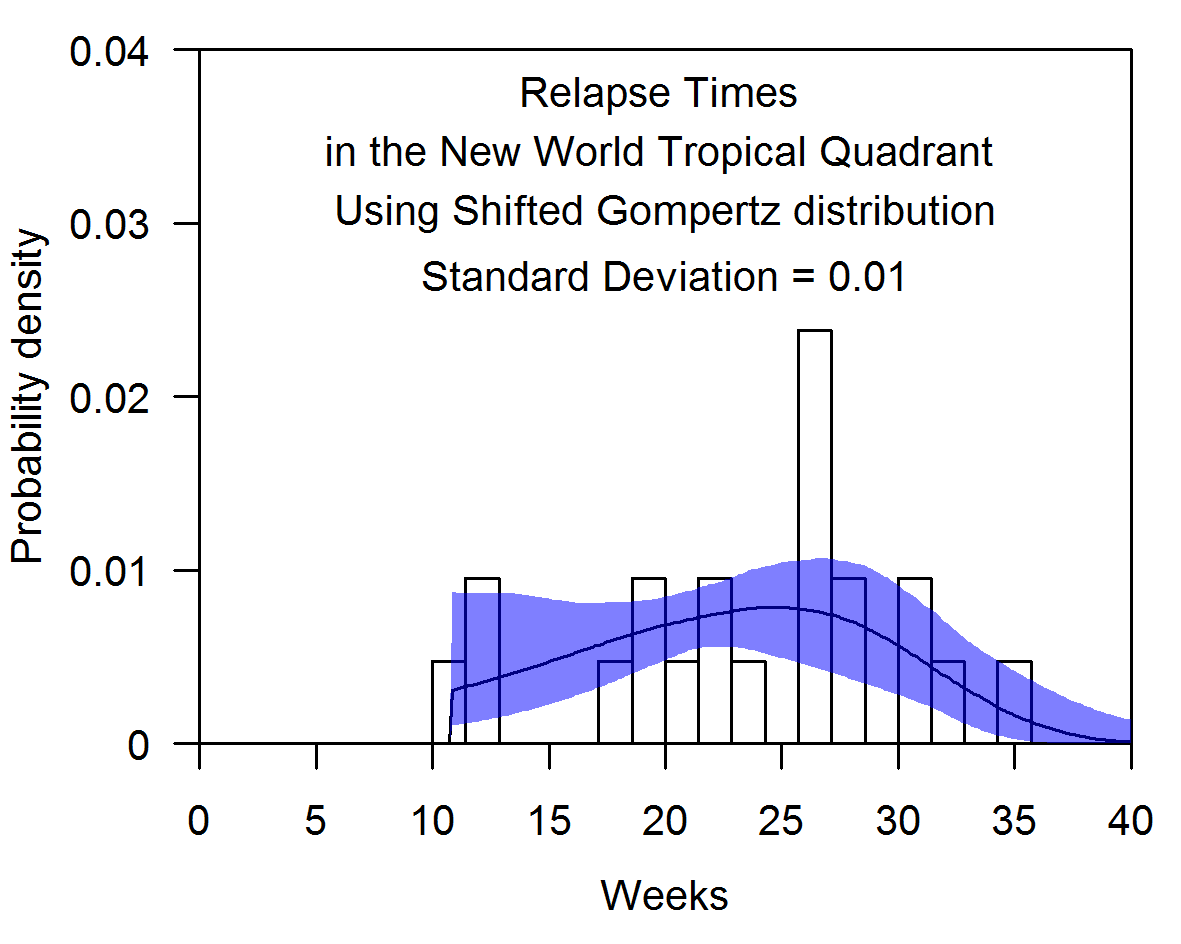


Figure S15. Sensitivity analysis of relapse period data (New World Tropical region) with 0.01 SD “noise” in dark blue

## Section II. Kaplan-Meier plots (incubation data)


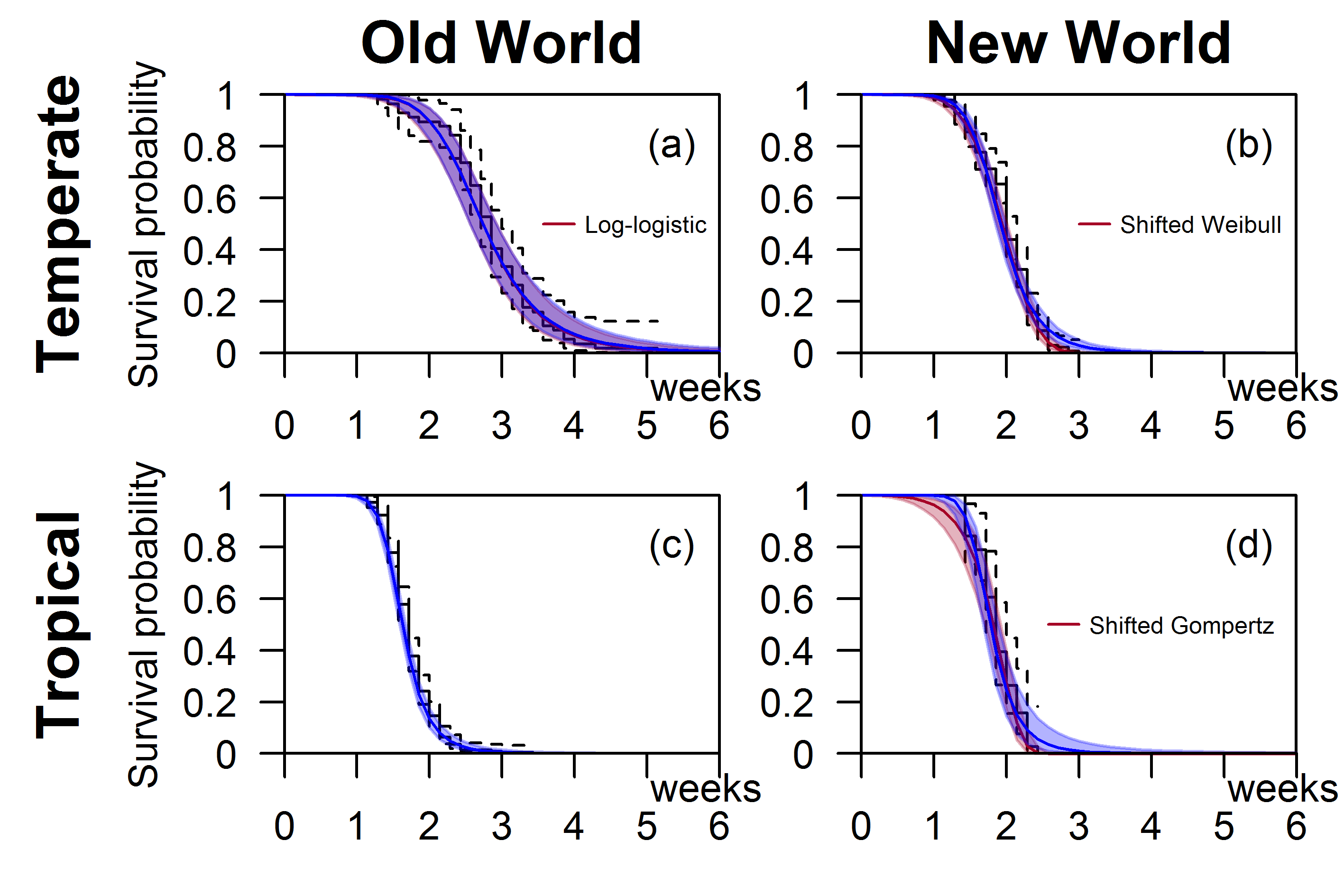


Figure S16. Comparison of best-fit shifted log-logistic distribution to Kaplan-Meier curves with 95% CIs for experimental incubation period data. Note: subregion-specific best-fit distributions in red.


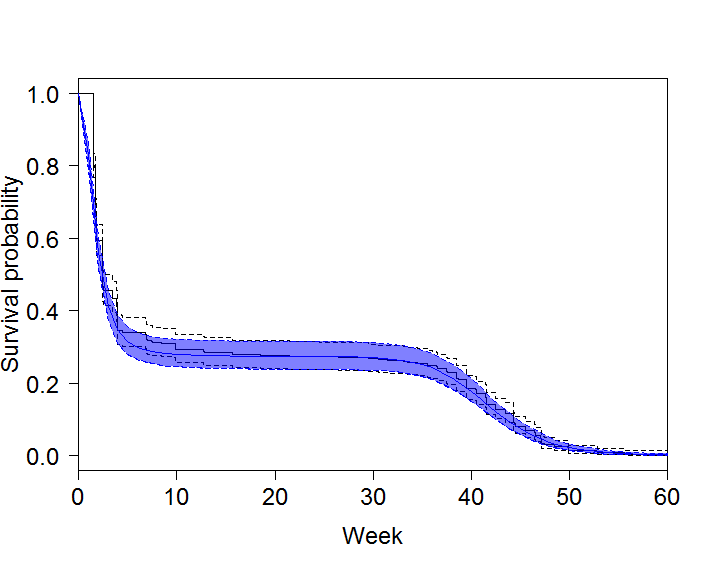


Figure S17. Comparison of best-fit mixture log-logistic distribution to Kaplan-Meier curves with 95% CIs for observational incubation periods.

## Section III. Kaplan-Meier plots and censoring (relapse data)


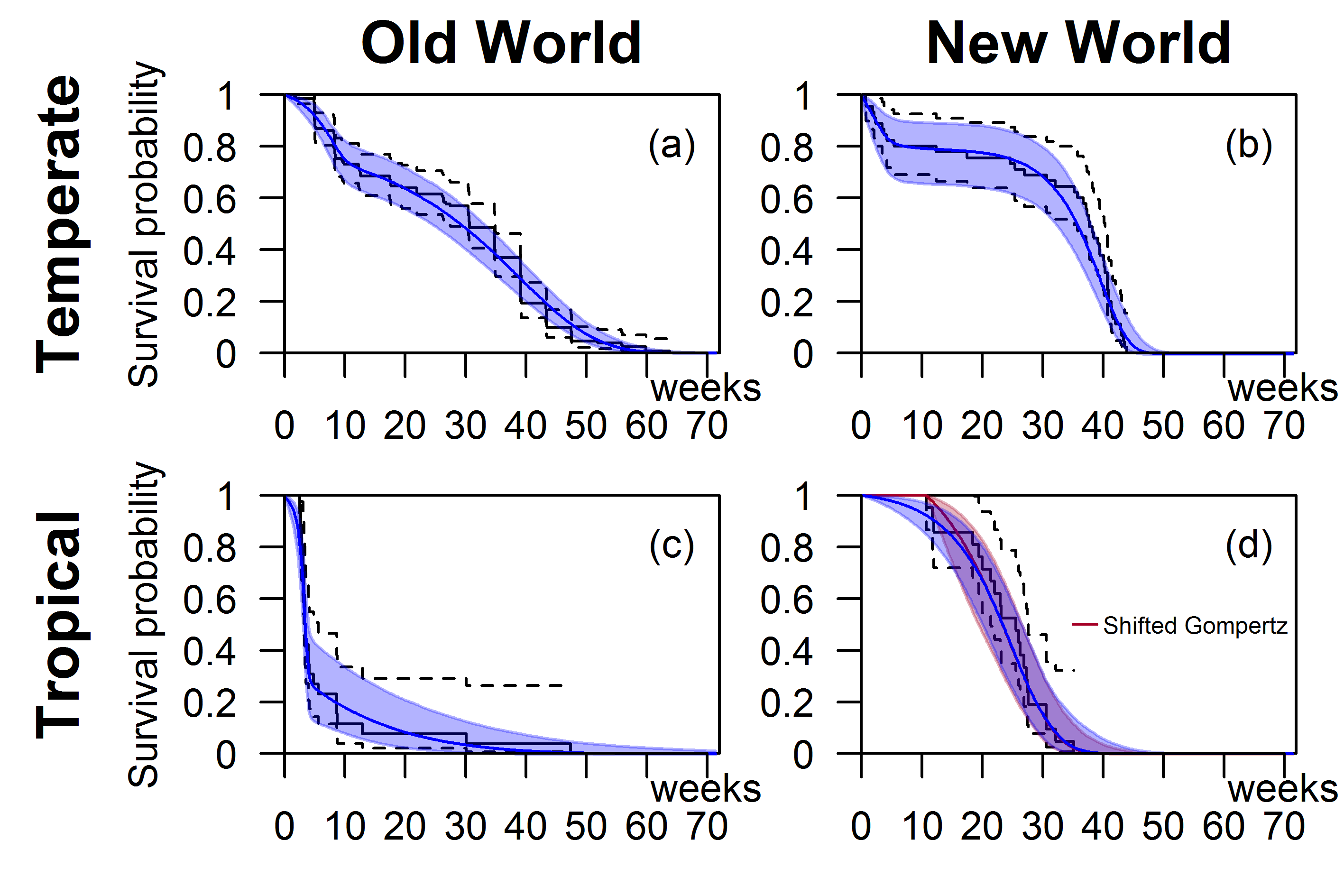


Figure S18. Comparison of best-fit mixture Gompertz distribution to Kaplan-Meier curves with 95% CIs for experimental relapse period data. Note: subregion-specific best-fit distribution in red.

The following section utilized Stata 13.1 (College Station, Texas, USA) for all analyses.

| **Region** | **N** | **censored (n)** | **censored (%)** | **95% CI** |
| --- | --- | --- | --- | --- |
| Old World, Tropical | 28 | 2 | 7.1 | 0.87 to 25.8 |
| New World, Tropical | 38 | 17 | 44.7 | 26.1 to 71.6 |
| Old World, Temperate | 206 | 76 | 36.9 | 29.1 to 46.2 |
| New World, Temperate | 48 | 3 | 6.3 | 1.3 to 18.3 |
|  |  |  |  |  |
| **All regions** | 320 | 98 | 30.6 | 24.9 to 37.3 |

Table S15. Censoring of crude (non-data augmented) full-cohort relapse data.

Figure S19. Kaplan-Meier plot, full-cohort relapse data (N=320).

Figure S20. Stratified Kaplan-Meier plot, full-cohort relapse data (N=320).

Figure S21. Kaplan-Meier plot, censored-cohort relapse data (N=222).

Figure S22. Stratified Kaplan-Meier plot, censored-cohort relapse data (N=222).

## Section IV. Parameterization of distributions

| **Distribution** | **parameters** | **function; R package** |
| --- | --- | --- |
| exponential | (rate) | (dexp; stats) |
| gamma | (shape, rate) | (dgamma; stats) |
| Gompertz | (shape, rate) | (dgompertz; flexsurv) |
| log-logistic | (shape, scale) | (dllog; FAdist) |
| lognormal | (meanlog, sdlog) | (dlnorm; stats) |
| Weibull | (shape, scale) | (dweibull; stats) |
| 3 parameter log-logistic | (shape, scale, shift) | (dllog3; FAdist) |
|  |  |  |

Table S16. Parameterizations utilized for model fitting in R software.
