# Supplementary figures and images for: The distribution of incubation and relapse times in experimental human infections with the malaria parasite Plasmodium vivax
Source: BMC Infect Dis. 2014 Oct 4;14:539. doi: 10.1186/1471-2334-14-539 (PMC4287165; doi:10.1186/1471-2334-14-539)

**A**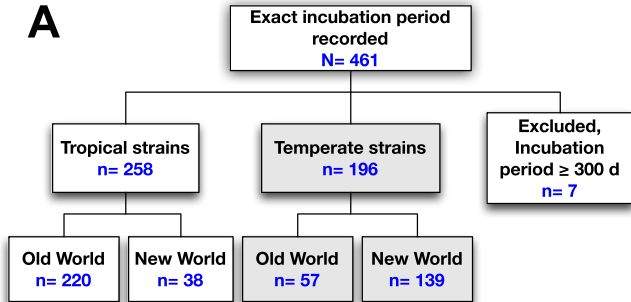**B**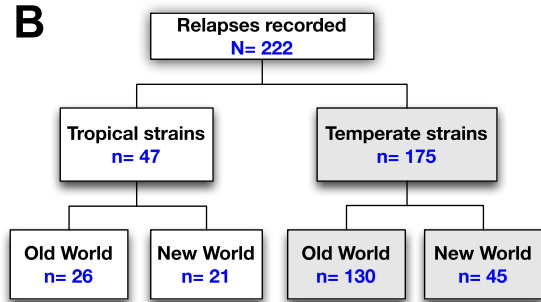

Supplement: Supplementary file 2 — Authors’ original file for figure 1 [file 12879_2014_3996_MOESM2_ESM.pdf]

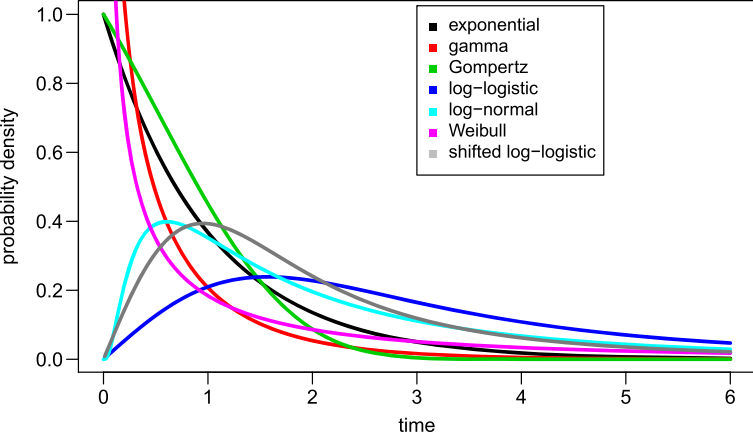

Supplement: Supplementary file 3 — Authors’ original file for figure 2 [file 12879_2014_3996_MOESM3_ESM.pdf]

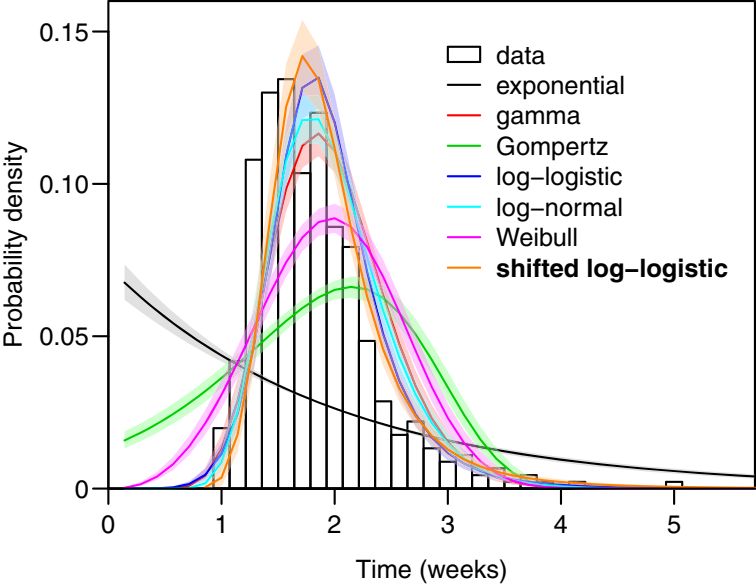

Supplement: Supplementary file 4 — Authors’ original file for figure 3 [file 12879_2014_3996_MOESM4_ESM.pdf]

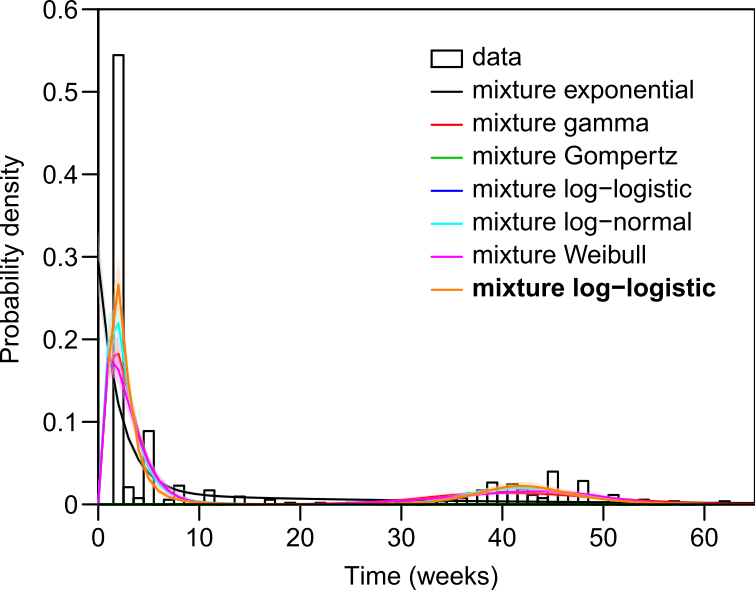

Supplement: Supplementary file 5 — Authors’ original file for figure 4 [file 12879_2014_3996_MOESM5_ESM.pdf]

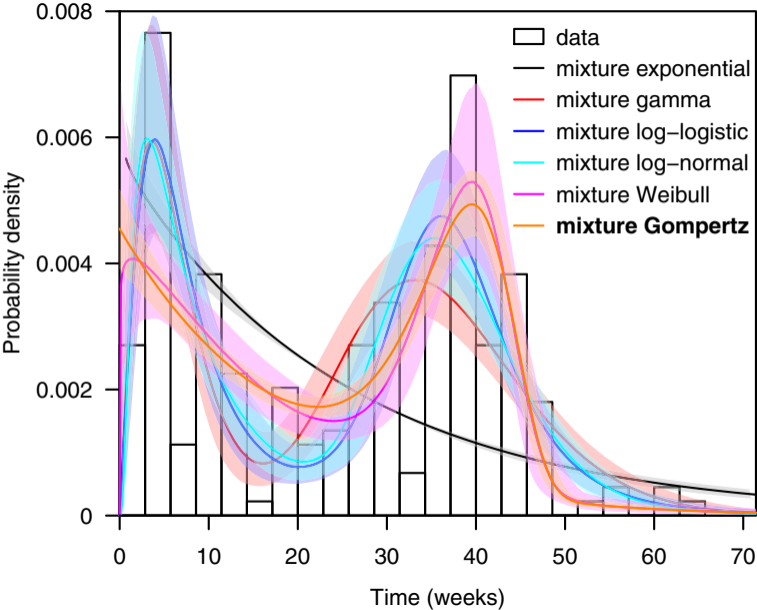

Supplement: Supplementary file 6 — Authors’ original file for figure 5 [file 12879_2014_3996_MOESM6_ESM.pdf]

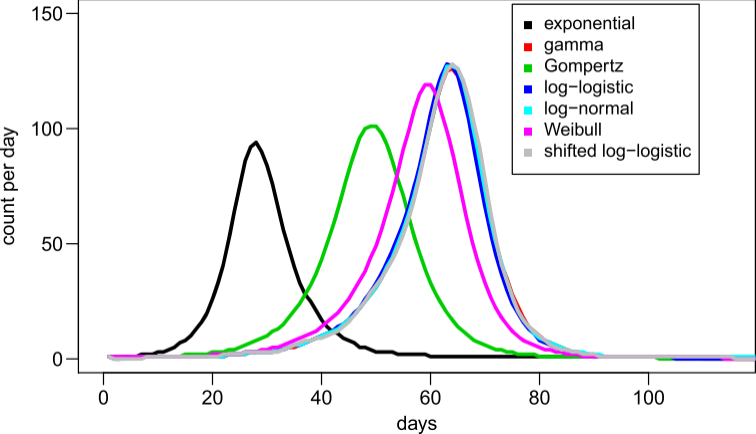

Supplement: Supplementary file 7 — Authors’ original file for figure 6 [file 12879_2014_3996_MOESM7_ESM.pdf]
